# A novel human tau knock-in mouse model reveals interaction of Abeta and human tau under progressing cerebral amyloidosis in 5xFAD mice

**DOI:** 10.1186/s13195-022-01144-y

**Published:** 2023-01-14

**Authors:** Susan Barendrecht, An Schreurs, Stefanie Geissler, Victor Sabanov, Victoria Ilse, Vera Rieckmann, Rico Eichentopf, Anja Künemund, Benjamin Hietel, Sebastian Wussow, Katrin Hoffmann, Kerstin Körber-Ferl, Ravi Pandey, Gregory W. Carter, Hans-Ulrich Demuth, Max Holzer, Steffen Roßner, Stephan Schilling, Christoph Preuss, Detlef Balschun, Holger Cynis

**Affiliations:** 1 Department of Drug Design and Target Validation, Fraunhofer Institute for Cell Therapy and Immunology, Weinbergweg 22, 06120 Halle, Germany; 2 KU Leuven, Faculty of Psychology and Educational Sciences, Brain & Cognition, Tiensestraat 102, box 3714, 3000 Leuven, Belgium; 3 Martin Luther University Halle-Wittenberg, Institute for Human Genetics, Magdeburger Strasse 2, 06112 Halle, Germany; 4 The Jackson Laboratory, 600 Main St, Bar Harbor, ME 04609 USA; 5 Paul Flechsig Institute for Brain Research, Leipzig University, Liebigstraße 19, 04103 Leipzig, Germany; 6 Anhalt University of Applied Sciences, Bernburger Straße 55, 06366 Köthen, Germany

**Keywords:** Alzheimer’s disease, Animal model, Tau, Knock-in, Amyloid, Gene expression, Synaptic function

## Abstract

**Background:**

Hyperphosphorylation and intraneuronal aggregation of the microtubule-associated protein tau is a major pathological hallmark of Alzheimer’s disease (AD) brain. Of special interest is the effect of cerebral amyloid beta deposition, the second main hallmark of AD, on human tau pathology. Therefore, studying the influence of cerebral amyloidosis on human tau in a novel human tau knock-in (htau-KI) mouse model could help to reveal new details on their interplay.

**Methods:**

We studied the effects of a novel human htau-KI under fast-progressing amyloidosis in 5xFAD mice in terms of correlation of gene expression data with human brain regions, development of Alzheimer’s-like pathology, synaptic transmission, and behavior.

**Results:**

The main findings are an interaction of human beta-amyloid and human tau in crossbred 5xFADxhtau-KI observed at transcriptional level and corroborated by electrophysiology and histopathology. The comparison of gene expression data of the 5xFADxhtau-KI mouse model to 5xFAD, control mice and to human AD patients revealed conspicuous changes in pathways related to mitochondria biology, extracellular matrix, and immune function. These changes were accompanied by plaque-associated MC1-positive pathological tau that required the htau-KI background. LTP deficits were noted in 5xFAD and htau-KI mice in contrast to signs of rescue in 5xFADxhtau-KI mice. Increased frequencies of miniature EPSCs and miniature IPSCs indicated an upregulated presynaptic function in 5xFADxhtau-KI.

**Conclusion:**

In summary, the multiple interactions observed between knocked-in human tau and the 5xFAD-driven progressing amyloidosis have important implications for future model development in AD.

**Supplementary Information:**

The online version contains supplementary material available at 10.1186/s13195-022-01144-y.

## Background

Alzheimer’s disease (AD) is a neurodegenerative disorder leading to progressive cognitive decline in elderly people [1]. The two main neuropathological hallmarks of AD are accumulation of amyloid beta (Aβ) in extracellular plaques and the formation of neurofibrillary tangles and neuropil threads resulting primarily from the aggregation of hyperphosphorylated protein tau [2].

Genetically engineered mice have been successfully used to model aspects of AD pathology, and these models are commonly applied in the development and testing process for novel treatment strategies [1–3]. First-generation models mainly overexpress human genes linked to familial forms of AD, aiming at an accelerated development of AD-like pathology. Thus, mutations in APP and PS1 are frequently used, which are derived from familial pedigrees of early-onset AD patients. However, high amyloid load alone does not induce tau pathology in available APP overexpressing mouse models [4]. Instead, to model tangle pathology, human MAPT gene mutations are used, which are not linked to AD but to tauopathies like frontotemporal dementia [5]. Furthermore, second-generation models aim at the expression of physiological levels of Aβ [6] and tau [7] resulting in novel insights, e.g., to tau biology [8].

A strong link on the molecular level has been established between Aβ and tau in first-generation mouse models. A crosstalk has been described for both molecules in several studies [9–11] establishing the necessity of tau in order for Aβ to exert its pathogenic properties [12, 13].

Despite high similarity between human and murine tau, there are also specific differences. It has been shown both in vitro and in vivo that the presence of mouse tau can inhibit the neurotoxic effects of human tau and human Aβ [14]. The most obvious discrepancy between humans and mice is related to tau splicing of exons 2, 3, and 10 [15, 16]. Thereby, exon 10 codes for an extra microtubule binding domain [15] leading to formation of tau with either three (3R) or four (4R) such domains. Both the 3R- and 4R tau isoforms are expressed at equal levels in human brain. Importantly, adult mice show only 4R expression [16, 17] but mouse tau is able to aggregate in vitro at similar levels as human tau [18, 19]. Therefore, the lack of tau pathology in mouse models could be due to a lack of 3R tau or due to other yet unidentified factors.

We engineered a novel human tau knock-in (htau-KI) mouse model by whole-gene replacement of murine tau by human tau [20] in parallel to a recently published htau-KI model [7]. Using crossbreds of htau-KI and 5xFAD mice, we studied the impact of high amyloid load on human wild-type (WT) tau. The examination of the 5xFADxhtau-KI mice in comparison to htau-KI littermates and controls at different ages allowed us to gain insights into the interplay between pathological levels of Aβ and physiological levels of human tau, and different methods identified a differential effect of human tau and murine tau under progressing cerebral amyloidosis.

## Methods

### Generation of htau-KI

**Fig. 1:**
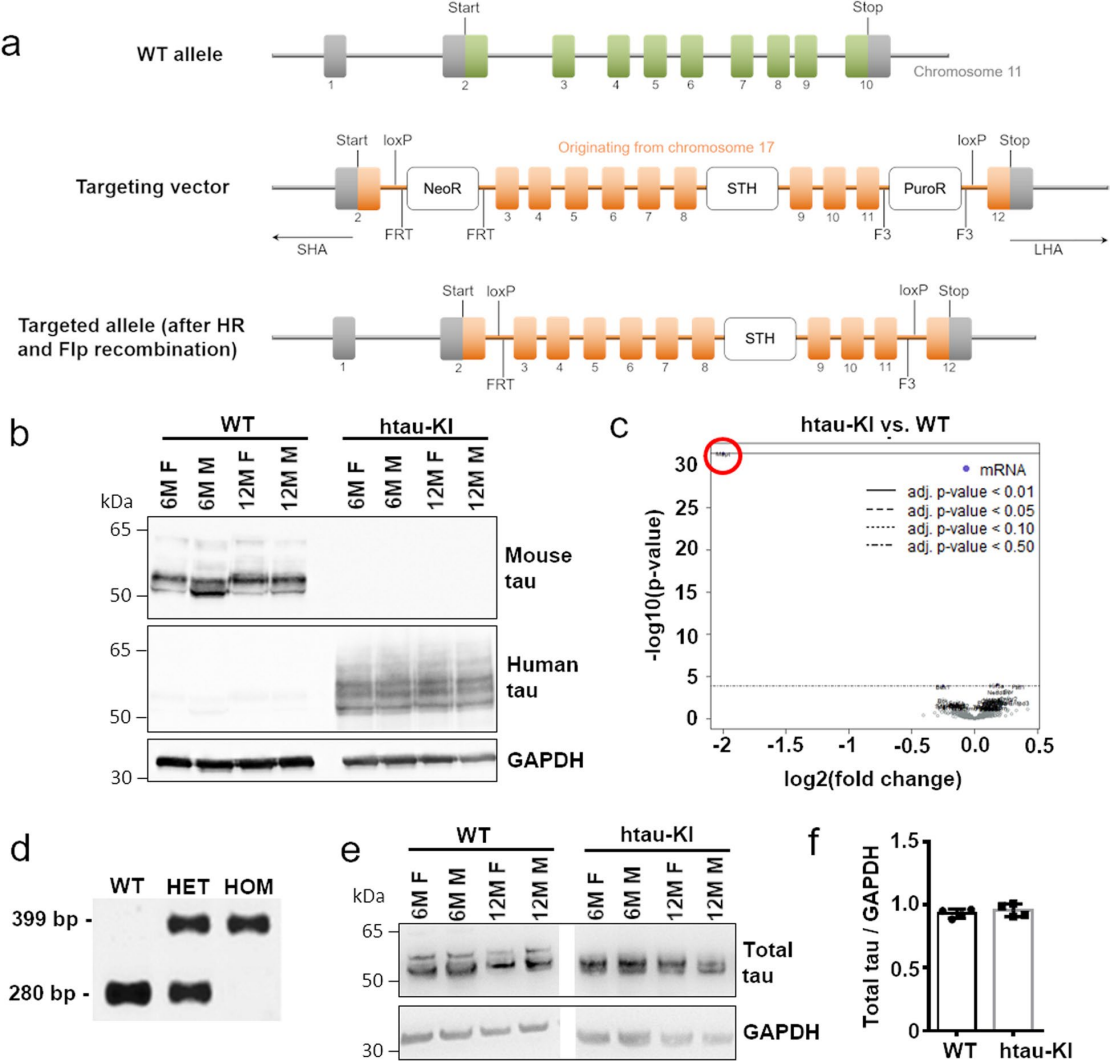
Generation of htau-KI mice. **a** Development strategy for the htau-KI mouse line. The human tau gene (exon 2–12, orange rectangles) was placed into the murine tau locus (green rectangles) by homologous recombination. The gene Saitohin (STH) is present in the human tau gene and was also included in the targeting vector. SHA = short homology arm, LHA = long homology arm, HR = homologous recombination, Flp = Flippase, F3 = Flp recombination target site, FRT = Flp recombination target site, NeoR = neomycin resistance gene, PuroR = puromycin resistance gene. **b** Mouse (anti-tau clone T49) and human (anti-tau13) tau-specific antibodies confirmed the absence of murine tau and the presence of human tau in dephosphorylated brain extracts of htau-KI mice, respectively. Faintly visible bands of human tau in WT mice are due to incomplete stripping of the membrane. M = male, F = female at ages of 6 and 12 months. The images have been cropped. Original files can be found as Additional files 4, 5 and 6. **c** Differential expression of 770 AD-related genes in frontal cortex of 13-month-old WT mice (*n*=6) vs. htau-KI (*n*=6) mice analyzed by nCounter Mouse AD gene expression panel. Depicted is a volcano plot showing log2(fold change) in dependence of −log10 of *P*-values. Red circle marks the only differentially expressed AD-related gene, MAPT. **d** Representative PCR using murine- and human tau-specific primers confirming the loss of the mouse tau gene and the presence of the human tau gene in homozygous htau-KI mice. Murine tau PCR product: 280 bp, human tau PCR product: 399 bp. Original image file can be found as Additional files 7. **e** A pan-specific tau antibody (Tau-5) on Western blot showed equal expression levels of tau in htau-KI mice compared to WT mice. M = male, F = female at ages of 6 and 12 months. Uncropped image files can be found as Additional files 8, 9, 10 and 11. **f** Quantification of total tau expression from **e** in WT and htau-KI mice with GAPDH as reference protein. Mean ± SD. *n*=4

The htau-KI mouse line was generated together with Taconic Bioscience. The bacterial artificial chromosome (BAC) RP11-111I23 containing the whole human tau sequence was used to generate a targeting vector, which has the mouse genomic sequence from the translation initiation codon in exon 2 to the termination codon in exon 10 replaced with its human counterpart. This vector was transfected into mouse ES cells from the C57BL/6NTac strain. Two ES cell lines, Mapt 20955-A-C08 and Mapt 20955-A-F07, were obtained. Line Mapt 20955-A-C08 resulted in offspring with a chimerism >50% after injection into blastocysts of BALB/c females. Blastocyst injection of line Mapt 20955-A-F07 did not result in chimeric offspring. Therefore, all generated mice resulted from ES cell line Mapt 20955-A-C08. Based on coat color, the mice with highest chimerism were selected and bred to C57BL6/NTac females. Germline transmission was confirmed by birth of black offspring. Mice were subsequently genotyped to verify the presence of the htau gene (See Fig. 1 for a scheme of the targeting strategy). Eight males and ten females were used as founders for establishment of the colony. Mice were backcrossed for two generations to C57BL/6J background and analyzed for a homozygous deletion in the Nnt gene as major hallmark of the C57BL/6J strain.

### Animals

All animal experiments were approved by the responsible animal ethics committee of the state of Saxony-Anhalt, Germany (Landesverwaltungsamt Sachsen-Anhalt, Department of Consumer Protection and Veterinary Affairs, Halle (Saale), Saxony-Anhalt, Germany) under the following approval number: 42502-2-1371 MLU. For electrophysiology, mice were bred in Halle, Germany, and transported to KU Leuven, Belgium, a minimum of 2 weeks ahead of testing. Housing and animal procedures were approved by the KU Leuven Ethical Committee (project P181/2017). We confirm that all animal handling procedures were carried out in accordance with directive 2010/63/EU of the European Parliament and of the Council on the protection of animals used for scientific purposes, the Belgian and German Animal Protection Acts and recommendations from the Federation of European Laboratory Animal Science Associations (FELASA).

After establishing the line, htau-KI^+/+^ and 5xFADxhtau-KI^+/+^ mice were generated from a mating between heterozygous 5xFADxhtau-KI^+/−^ mice and homozygous htau-KI^+/+^ mice. C57BL/6J WT controls and 5xFAD mice were obtained by mating 5xFAD mice with C57BL/6J WT mice. All mice were kept on a C57BL/6J background, and all 5xFAD mice were bred heterozygous. Age-matched phenotyping groups of 6 and 12 months were generated using timed mating. Mice had access to water and food ad libitum and were kept on a regular 12/12-h light/dark cycle. After concluding behavioral analyses, the mice were sacrificed at an age of 7 and 13 months, respectively.

### Genotyping

At the age of 19–21 days, ear punches were taken to individually mark the mice. The resulting material from each mouse was used for genotyping. After sacrificing the mice at the end of the respective studies, tail tips were taken for confirmation of the genotype. DNA was isolated from the ear punches/tail tips using the 1-Step kit (Nexttec, Leverkusen, Germany) according to the manufacturer’s protocol. The DNA was analyzed for the presence of 5xFAD and htau genes using the GoTaq DNA Polymerase (Promega, Madison, Wisconsin). Primers were specific for the different genes, and sequences are shown in Supplementary Tab. 4. Samples were afterwards loaded onto a 1.6% agarose gel in TAE buffer with ethidium bromide to visualize the PCR products.

### Organ collection

After completion of all behavioral experiments, mice were euthanized using CO_2_ inhalation and subsequently perfused with 20 mL PBS. The brain was collected and divided into its two hemispheres; the left hemisphere was fresh frozen on dry ice, and the right hemisphere fixed in 4% PFA. All fresh frozen samples were subsequently stored at −80°C.

### Biochemical analyses

Fresh frozen left-brain hemispheres were homogenized in cell extraction buffer (Invitrogen) using the following protocol: frozen brain was transferred to a Dounce tissue homogenizer, after which 1 mL of cell extraction buffer (Invitrogen, Carlsbad, California) was added. Brain tissue was mechanically homogenized, followed by incubation on ice for 30 min. Every 10 min, the homogenate was vortexed and resuspended. Subsequently, the samples were centrifuged for 30 min at 13,000×*g* and 4°C. The supernatant was collected in a fresh Eppendorf tube, and both supernatant and pellet were stored at −20°C until further use. 

### Western blot

Brain homogenates were thawed, and protein concentration was measured using the Pierce Bradford assay (Thermo Fisher Scientific) according to the manufacturer’s protocol. Selected brain extracts were also dephosphorylated using lambda phosphatase essentially as described elsewhere [7]. SDS-PAGE was performed using the NuPage system and reagents (Invitrogen), following the protocol of the manufacturer. The Pageruler™ Plus Prestained Protein Ladder, 1.0 mm Bis-Tris gels with 12 or 17 wells, and MES SDS running buffer were used. Dephosphorylated tau was analyzed on 12.5 % SDS-PAGE Tris gels. For subsequent Western blotting, the semi dry system by Galileo Biosciences (Cambridge, Massachusetts) was used according to the manufacturer’s instructions. Proteins were blotted on 0.2 μm nitrocellulose membranes (Amersham Biosciences, Little Chalfont, UK) for 90 min at 38 mA. Afterwards, the membrane was blocked while floating for 30 min in a box with 5 % milk 0.05 % Tween TBS, under continuous shaking. The membrane was then placed in a 50-mL tube and incubated over night with the primary antibody (see Supplementary Tab. 3; dissolved in 5 % milk 0.05 % Tween TBS) at 4 °C on a roller bank. The following day, the membrane was washed three times 5 min in 0.05 % Tween TBS, after which it was incubated with the secondary antibody (see Supplementary Tab. 3), also diluted in 5 % milk 0.05 % Tween TBS (1 h, RT, on roller bank). Subsequently, the membrane was washed twice in 0.05 % Tween TBS, and once in TBS (all washes 5 min, RT, on a roller bank). For protein detection, the SuperSignal West Pico or Femto kit (Thermo Fisher Scientific) was used according to the manufacturer’s protocol. Bio1D software (Vilber Lourmat, Collégien, France) or ImageJ was used for quantification of signal intensity. For absolute quantification of 3R and 4R tau, the tau protein ladder (Sigma Aldrich, St. Louis, MO, USA) has been used as a standard. Protein amounts were calculated densitometrically in ImageJ by comparing GAPDH normalized signal intensities of samples with bands of the tau ladder.

### RNA isolation and qRT-PCR

After removal of the olfactory bulb, a small piece of prefrontal cortex was removed from the fresh frozen left-brain hemisphere for RNA isolation. The Nucleospin RNA kit (Macherey Nagel, Oensingen, Switzerland) was used according to the manufacturer’s protocol with some minor adjustments. TCEP was used instead of β-mercaptoethanol in the lysis step. Furthermore, RNA was eluted in 40-μL RNase-free H_2_O instead of 60 μL, and the eluate was loaded onto the spin column a second time to retrieve higher RNA yield. RNA concentrations were measured using a Nanodrop 2000 (Thermo Fisher Scientific). The cDNA was synthesized using the Superscript II kit (Invitrogen), according to the manufacturer’s protocol. Three hundred nanograms total RNA was used, and random primers were added during the first step.

A qRT-PCR was performed for quantification of 3R and 4R tau isoforms. Therefore, a standard curve of double-stranded DNA (Metabion, Planegg, Germany) was prepared. The sequences were specific for 3R and 4R and are shown in Supplementary Tab. 4. A standard curve from 30 to 300,000 molecules/μL was prepared. Two biological replicates of the standard curve were prepared, and the standard curve best fitting a linear equation (*R*^2^ > 0.98) was used to calculate the DNA concentration of the other samples. Primers were specific for 3R and 4R tau isoforms, as described by Ingelsson et al. [21]. The specificity was confirmed by a lack of signal when 3R primers where used with 4R tau DNA and vice versa. All primer sequences are shown in Supplementary Tab. 4. The Rotorgene 3000 system (Corbett research, Mortlake, Australia) was used, and the SYBR Green kit (Applied Biosystems, Foster City, California) according to the manufacturer’s protocol. For each biological replicate, three technical replicates were performed, and a maximum difference of 0.4 CT was allowed between technical replicates.

### Gene expression analysis of AD-specific genes

Isolated RNA of 12 mice per genotype (7- and 13-month-old males and females, three each) was used for the nCounter Mouse AD gene expression panel developed by NanoString Technologies (Seattle, Washington). One hundred to one hundred fifty nanograms of RNA was used to set up the hybridization reaction, after which the experiment was performed according to the manufacturer’s protocol. The prep station was run with high-sensitivity settings, and the Analyzer with MAX data resolution (555 FOV). Data normalization and analysis was performed using the nSolver 4.0 software (NanoString Technologies) according to the manufacturer’s protocol. Normalization was done by dividing counts within a lane by geometric mean of the housekeeping genes from the same lane. For the downstream analysis, counts were log-transformed from normalized count values.

#### Mouse-human co-expression data comparison

Data on 30 harmonized human co-expression modules, significantly enriched for differentially expressed genes in AD post mortem brain samples, were obtained via the AD Knowledge Portal (https://www.synapse.org/#!Synapse:syn11914606). The data derives from over 2000 human brain samples from three independent AD cohorts (ROSMAP, Mayo Clinic, Mount Sinai Brain Bank) and includes seven distinct brain regions. Details on post mortem brain sample collection, tissue and RNA preparation, and sequencing can be found elsewhere [22–24]. Among the 30 human co-expression modules, five specific consensus clusters have been previously described by Wan et al. [25]. These consensus clusters consist of overlapping co-expression modules, which are associated with specific AD-related changes that were highly preserved across studies and brain regions. In order to align human with mouse data, a Nanostring gene expression panel in combination with a recently described systems biology approach was used [26]; https://www.synapse.org/#!Synapse:syn23569889) to assess LOAD-relevance of the novel mouse models. Briefly, gene expression changes (log fold change) across a set of human key genes within AD relevant co-expression modules were correlated with expression changes in the novel mouse model to determine opposing and concordant effects driven by htau and amyloid. To obtain log fold changes associated with htau in the htau-KI and amyloid effects in the 5xFAD model, differential gene expression analysis was performed for each mouse model and sex using the voom-limma package in R [27]. All log fold change values for human transcripts were obtained via the AD Knowledge Portal as previously reported (https://www.synapse.org/#!Synapse:syn23569889). The correlation between changes in expression (log fold change) was computed for each gene in a given expression module with each mouse model, sex, and age. Correlation coefficients were obtained using cor.test function built in R as: cor.test( LogFC(h), LogFC(m)), where LogFC(h) is the log fold change in transcript expression of human AD patients compared to control patients and LogFC(m) is the log fold change in expression of mouse transcripts compared to control mouse models. Significance of cross species post mortem brain module correlations were determined after FDR multiple testing correction, and age-, strain-, and sex-specific effects were assessed using a multiple linear regression analysis.

### Gene set enrichment analysis in mouse and human data

Gene set enrichment analysis based on the method proposed by Subramanian et al. [28] was performed using the cluster profiler package in the R software environment [29] for the Kyoto Encyclopedia of Genes and Genomes (KEGG) database. Briefly, human data with log fold changes for the seven AMP-AD brain regions were obtained through the AD Knowledge Portal (https://www.synapse.org/#!Synapse:syn14237651). Only orthologous genes on the NanoString Mouse AD panel were selected and KEGG pathway enrichment was performed for each brain region independently to identify significantly up- and downregulated gene sets. For the mouse data, differential expression analysis between the 5xFAD and 5xFADxhtau-KI models was performed to obtain a list of fold changes highlighting genes that are either up- or downregulated in the presence of htau on the 5xFAD background. Enrichment scores for all significantly associated KEGG pathways were computed to compare relative expression on the pathway level between post mortem brain samples and the 5xFADxhtau-KI mouse model. Finally, we extracted the leading-edge gene set, which identified the genes that appeared in the ranked list at or before the point, at which the running sum reaches its maximum deviation from zero. This leading-edge subset can be interpreted as the core that accounts for the gene set’s enrichment signal.

### Immunohistochemistry

The right hemisphere was fixed in 4% PFA for 2 days at 4°C, followed by incubation in 30% sucrose solution at 4°C. After a minimum of 2 days, the organs were washed in PBS, and subsequently frozen in Tissue-Tek O.C.T. (Sakura Finetek, Alphen aan den Rijn, the Netherlands) in plastic molds. Sagittal slices (30 μm) of the hemisphere were prepared using a cryomicrotome (Cryostar NX70, Thermo Fisher Scientific, Waltham, Massachusetts), starting in the center of the brain. Slices containing the hippocampal formation were collected in 24-well plates and stored at 4°C in PB buffer containing 0.025% sodium azide until staining.

For free-floating 3,3’-diaminobenzidine (DAB) staining, the slices were washed in TBS (5 min, RT), after which the endogenous peroxidase was blocked by incubation with 1% H_2_O_2_ in 60% MeOH (30 min, RT). Subsequently, the slices were washed three times in TBS, followed by blocking with 5% goat serum in 0.3% Triton-X TBS (30 min, RT). For mouse-on-mouse staining, M.O.M. reagent (Vector Laboratories, Burlingame, California) was added during the blocking step. The primary antibodies (see Supplementary Tab. 3) were diluted in 5% goat serum 0.1% Triton-X TBS, and the slices incubated overnight at 4°C in a humidified chamber. The following day, slices were washed in TBS three times, followed by incubation with the respective secondary antibodies (see Supplementary Tab. 3), which were diluted in 2% BSA TBS (1h, RT). After another three washes in TBS, slices were incubated with ExtrAvidin® Peroxidase, diluted 1:1,000 in 2% BSA TBS (1h, RT), followed by two times washing in TBS, and one time in Tris buffer. DAB staining was performed by incubating slices in 0.05% DAB 0.015% H_2_O_2_ in Tris buffer for 4 min (Iba-1, GFAP, 3A1) or 8 min (all other applied antibodies) at RT. Subsequently, the slices were washed once in Tris buffer and once in TBS, followed by placing the slices on glass slides, coated with protein glycerol (Carl Roth, Karlsruhe, Germany). After the sections were air dried, the slides were dehydrated by immersing them twice in ethanol followed by immersion in ROTI®-Histol (Carl Roth). Finally, the slices were embedded using Permount mounting media (Fisher Scientific, Hampton, New Hampshire) and closed with cover slips. Slides were stored at RT.

After air drying, Congo red amyloid staining was performed according to Uhlmann et al. [30]. In brief, sections were incubated in alkaline saturated NaCl solution for 20 min followed by incubation in filtered alkaline Congo red solution (0.2 %) for 30 min at room temperature. After dipping the slides eight times in 95% ethanol and 2 × 30s in 100% ethanol the sections were dehydrated and embedded as described above. Slices were recorded with a BZ-9000E microscope (Keyence, Osaka, Japan), and subsequent quantification of plaques was performed manually or using the BZ-II-Analyzer software (Keyence).

### Electrophysiology

#### Acute slice preparation

Mice were sacrificed by cervical dislocation, after which the brain was rapidly dissected and immersed in ice-cold artificial cerebrospinal fluid (ACSF), saturated with carbogen (95% O_2_, 5% CO_2_) and containing (all in mM) 124.0 NaCl, 4.9 KCl, 1.3 MgSO_4_, 2.5 CaCl_2_, 1.2 KH_2_PO_4_, 25.6 NaHCO_3_, and 10.0 glucose (pH 7.4). From the left hemisphere, the hippocampus was immediately isolated and cut into 300-μm-thick slices using a custom-made tissue chopper for field potential recordings. The part of the right hemisphere containing the medial hippocampus was cut into 400-μm-thick slices using a vibratome (Microm HM 650 V, Thermo Fisher Scientific) for whole-cell recordings. The ages of the mice used for electrophysiology were (mean ± SD) as follows: 5xFAD = 8.3 ± 1.7 months, htau-KI = 7.6 ± 0.9 months, 5xFADxhtau-KI = 7.7 ± 1.1 months, WT = 8.1 ± 1.7 months.

#### Extracellular recording of field potentials

Hippocampal slices were placed in an incubation chamber (Warner Instruments, Hamden, Connecticut) with carbogenated ACSF (same composition as during slice preparation) and left to recover at RT for at least 1 h. For the recording of extracellular field potentials, a multi-electrode array (MEA) system (Multi Channel Systems, Reutlingen, Germany) was used as described before [31]. After incubation, a single slice was placed on a MEA chip (60 TiN electrodes in 8×8 layout and 100 μm spacing; imec, Heverlee, Belgium) [32] and perfused with ACSF at 2.5 mL/min and a constant temperature of 32°C. Stimulation and recording was performed using a stimulus generator (SG 4002), MEA1060-BC amplifier, temperature controllers (TC, PH01), and software (MEA_Select, MC_Stimulus, MC_Rack) from Multi Channel Systems. A single electrode located at the level of the Schaffer collaterals in the CA1 region was selected for biphasic constant voltage stimulation. The analysis of the evoked field excitatory post-synaptic potentials (fEPSPs) was focused on the signals from the electrode adjacent to the stimulation electrode (in anterograde direction of the Schaffer collaterals). Data streams were sampled at 10 kHz. First, an input/output curve was established using stimulation intensities ranging from 0.5 to 4 V (at 0.5 V intervals). The intensity leading to 35% of the maximum of the input/output curve was used for all subsequent stimulations. Next, a series of paired-pulse stimulations with intervals of 10, 20, 50, 100, 200, and 500 ms was applied. Last, after obtaining a stable baseline for at least 30 min (3 sweeps with 15 s interval, repeated every 3 min), LTP was induced by 3x theta-burst stimulation (TBS) (each TBS consists of 10 trains of 4 pulses at 100 Hz, with 200-ms intervals and 200 μs pulse-width; 10-min interval between subsequent TBS). fEPSPs were recorded for at least 120 min after LTP induction (3 sweeps with 15 s interval, repeated every 5 min). For analysis, raw data were extracted using MC_Rack software in replayer mode (Multi Channel Systems). fEPSP slope values were obtained with the region of interest set from peak-to-peak (10–90%), and all groups of 3 subsequent sweeps were averaged. Paired-pulse ratios were calculated by dividing the slope of the second fEPSP by the slope of the first. LTP recordings were normalized to the average baseline slope.

#### Whole-cell recordings

After cutting, slices were placed in an incubation chamber (Warner Instruments) with carbogenated ACSF (all in mM: 124.0 NaCl, 4.9 KCl, 1.2 NaH_2_PO_4_, 25.6 NaHCO_3_, 2.0 CaCl_2_, 2.0 MgSO_4_, 10.0 glucose, pH 7.3–7.4) and left to recover at RT for at least 90 min. Whole-cell voltage clamp recordings of CA1 pyramidal cells were performed at RT using a MultiClamp 700B patch-clamp amplifier and pClamp™ software (Molecular devices, San José, California), as previously described [33]. Recording electrodes, pulled from borosilicate glass (World Precision Instruments, Sarasota, Florida), were filled with a solution containing the following (in mM): 135 CsMeSO_4_, 4 NaCl, 4 Mg-ATP, 0.5 EGTA-Na, 0.3 Na-GTP, 10 K-HEPES, 5 QX-314; pH 7.3 (pipette resistance 3–5 MΩ). Access resistance was 10–20 MΩ and was then compensated to 75%. If the input resistance changed more than 25% during the recordings, the neuron was excluded from the study. Miniature excitatory and inhibitory post-synaptic currents (mEPSCs and mIPSCs) were measured, mostly consecutively from the same neurons. First, mEPSCs were recorded at the reversal potential for GABA_A_ receptor-mediated events (−60 mV), after which mIPSCs were recorded at the reversal potential for glutamatergic currents (+10 mV) with tetrodotoxin (1 μM) present in the bath medium. To verify that mEPSCs were indeed glutamatergic, they were blocked at the end of the experiments by applying 20 μM 6-cyano-7-nitroquinoxaline-2,3-dione (CNQX) and 10 μM d-aminophosphonovalerate (d-APV). Similarly, mIPSCs were verified by blocking them using 100 μM picrotoxin, a GABA_A_ receptor antagonist. Data were low-pass filtered at 2 kHz and acquired at 10 kHz using Digidata 1440 and pClamp™ 10 software. Offline analysis of mEPSCs and mIPSCs was performed using MiniAnalysis software (v.6.0.7, Synaptosoft, Decatur, Georgia).

### Behavioral studies

Mice were housed on a light regime of 12 h dark and 12 h light with lights on at 7 AM. Animals subjected to behavioral experiments were tested in the light phase of the day. The experimenter was blinded to the genotype of the mice for the complete duration of the tests, as well as the subsequent analysis of the videos.

#### Primary screening

Before starting full behavioral analysis, all mice were screened for general health according to the SHIRPA protocol [34]. Vision, hearing, grip strength and mobility were tested. The weight of each mouse was also documented.

#### Elevated plus maze

The setup was a plus shaped maze with arms of 40 cm × 7.5 cm, placed 70 cm above the ground. Two of the arms had walls with a height of 20 cm (closed arms), the other 2 arms were without walls (open arms). Mice were placed in one of the closed arms and allowed to explore freely for 10 min. Behavior was recorded with a camera attached to the ceiling above the maze and analyzed using Biobserve Viewer software (Biobserve GmbH, Bonn, Germany). The time spent in open arms was used as measure for exploratory and anxiety-like behavior.

#### Morris water maze

The Morris water maze consisted of a round pool (diameter 120 cm) with white walls, filled with water. A platform (diameter 10 cm) covered with white sports tape (for grip) was placed inside, 8 mm below water level. The water temperature was 26°C (±0.5°C). Four differently colored/shaped 3D environmental cues were attached to the side of the pool, to allow the mice to orientate. The protocol consists of 4 days, with 4 swimming trials each day. For each trial, a mouse was placed in the pool and allowed to swim and to find the platform within 60 s. If the mouse did not find the platform within 60 s, the experimenter led the mouse to the platform. When the mouse was on the platform, it was allowed to sit there for 10 s to orientate. The mice were subsequently dried with paper towels and warmed under a heat lamp for 5 min, followed by the next trial. Each trial, the mouse started in a different quadrant of the pool. The platform was always located in quadrant 3. During trials 1 and 4 of each test day, the mice started in quadrant 1, opposite from the platform. In the second trial, the mice started in quadrant 2, and in the third trial in quadrant 4. Movement of the mice was recorded using a camera, and the animal was tracked using the Biobserve Viewer software (Biobserve GmbH).

### Statistical analysis

Statistical analysis was done using GraphPad Prism 8 (GraphPad Software). One-way ANOVA with Tukey post hoc analysis was used. For electrophysiology, time series were tested in GraphPad Prism 8 using two-way repeated measures analysis of variance (RM-ANOVA) and Tukey’s or Dunnett’s multiple comparisons test. Because of significant differences in variance between samples of mEPSCs and mIPSCs, they were analyzed by Welch’s one-way ANOVA test using the two-stage linear step-up procedure of Benjamini, Krieger, and Yekutieli to correct for multiple testing. Cumulative distribution functions of mEPSCs and mIPSCs were compared by the Kolmogorov-Smirnov test. For analysis of Morris water maze data, RM-ANOVA were performed using the lmerTest package in R with Tukey post hoc test. For the gene expression analysis of the NanoString Mouse AD panel, counts were log-transformed from normalized count values. Differential gene expression analysis was performed for each mouse model and sex using the voom-limma package in R. For the human to mouse comparison, the correlation between changes in expression (log fold change) across species was computed for each gene in a given AMP-AD expression module with each mouse model, sex, and age based on an established systems biology method to assess disease relevance of novel LOAD mouse models [26, 35, 36]. Briefly, Pearson’s correlation coefficients based on log fold changes differences were obtained for all co-expression module comparisons between human and mouse. Significant positive or negative correlations (FDR-adjusted *P* < 0.05) across all orthologous genes within each of the 30 human AMP-AD expression modules were assessed after multiple testing correction for all modules using the cor.test function built in R. Correlation plots were visualized using the corrplot package [37]. Gene set enrichment analysis was performed using the clusterprofiler package in R for the KEGG pathway database.

## Results

### Characterization of htau-KI and 5xFADxhtau-KI

Htau-KI mice were generated by electroporating the targeting vector (Fig. 1a) into C57BL/6 NTac ES cells followed by steps described in detail in the “Methods” section. Murine (T49) and human (Tau13) tau-specific antibodies confirmed the absence of murine tau and the presence of human tau at the protein level in htau-KI mice, respectively (Fig. 1b). Furthermore, mRNA analysis using NanoString technology and PCR corroborated the absence of murine tau expression in htau-KI mice (Fig. 1c, d) and 5xFADxhtau-KI crossbreds (Supplementary Fig. 1b). We found comparable expression levels of human tau in htau-KI mice and endogenous murine tau in WT mice by Western blotting using a pan-tau-specific antibody (Tau-5) (Fig. 1e). Quantification of the protein expression level by normalizing to GAPDH expression confirmed this finding (Fig. 1f).

**Fig. 2:**
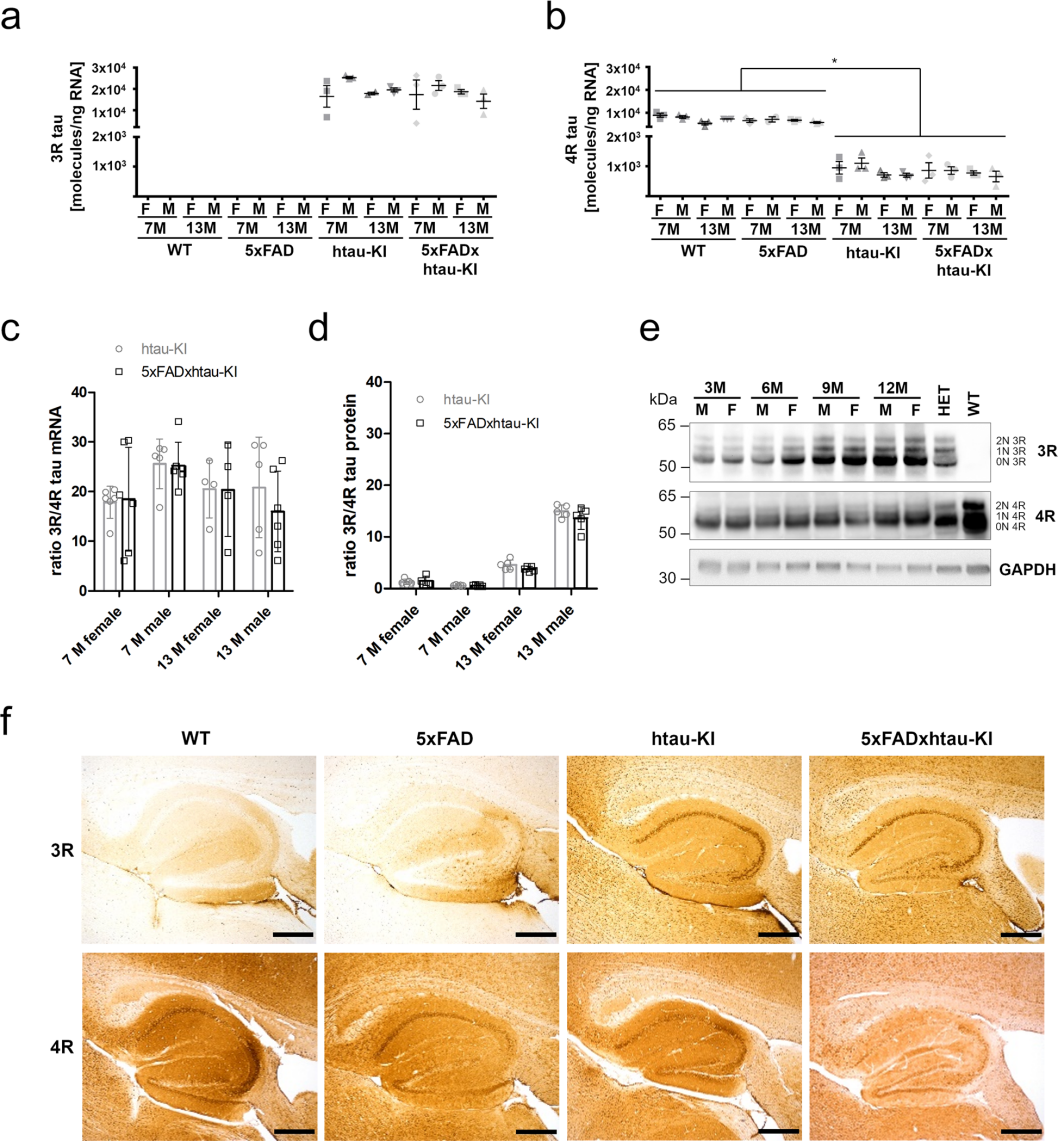
Analysis of tau isoform expression in htau-KI mice and generated crossbreds. **a**, **b** Absolute quantification of 3R tau (**a**) or 4R tau (**b**) based on a standard curve generated from a human 3R tau- or 4R tau-specific DNA sequence, respectively. Depicted are the amounts of 3R or 4R tau mRNA in molecules/ng RNA from male (M) and female (F) WT, 5xFAD, htau-KI and 5xFADxhtau-KI mice at the ages of 7 and 13 months. Note, no PCR product was obtained for WT and 5xFAD for 3R tau. Mean±SEM, *n* = 2–3 per group. *P < 0.05, one-way ANOVA, Tukey’s multiple comparison test. **c** Combined scatter and bar plot (mean±SD) for illustration of the 3R/4R tau mRNA ratio in htau-KI and 5xFADxhtau-KI mice depending on sex and age (7 and 13 months). Shown is a summary of 2 independent experiments. *n* = 4–6. **d** Combined scatter and bar plot (mean±SD) for illustration of the 3R/4R tau protein ratio in htau-KI and 5xFADxhtau-KI mice depending on sex and age (7 and 13 months). *n* = 5. **e** Western blots stained with 3R- and 4R-specific antibodies in male (M) and female (F) htau-KI mice at the ages of 3–12 months in comparison to the reference protein GADPH. WT mice only express 4R tau isoforms. Tau isoforms are labeled on the right. Het = heterozygous htau-KI mouse, xM = age in months. Uncropped image files can be found as Additional files 12, 13 and 14. **f** Immunohistochemical staining using 3R- and 4R-specific antibodies. Depicted are representative images of the hippocampal structure from female WT, 5xFAD, htau-KI, and 5xFADxhtau-KI mice at 13 months of age. Note the presence of 3R tau in pyramidal neurons in htau-KI and 5xFADxhtau-KI mice. The staining is representative of all sexes. Scale bar 500 μm

Mice and humans show differential expression of tau isoforms. Human tau is spliced into 6 major isoforms possessing either three (3R) or four (4R) microtubule binding domains. Adult mice express only three 4R tau splice isoforms [16] and lack 3R tau. To study 3R/4R ratios in the htau-KI and 5xFADxhtau-KI mice, we quantified 3R and 4R tau mRNAs and protein levels (Fig. 2). Using a qRT-PCR protocol for absolute quantification of 3R- and 4R tau, we confirmed the absence of 3R tau expression in WT and 5xFAD mice (Fig. 2a). In contrast, high expression levels of 3R tau mRNA were observed in htau-KI and 5xFADxhtau-KI (Fig. 2a). Murine 4R tau mRNA is highly expressed in WT and 5xFAD, whereas statistically significant lower levels of human 4R tau mRNA are seen in htau-KI and 5xFADxhtau-KI (Fig. 2b). There was no significant age-, sex-, or genotype-related shift in 3R- and 4R mRNA expression in the tested genotypes (Fig. 2a, b). Based on the determined tau molecules/ng RNA, the 4R/3R ratio revealed an approx. 20-fold higher gene expression of 3R tau compared to 4R tau in htau-KI and 5xFADxhtau-KI (Fig. 2c). Interestingly, for 3R/4R tau protein, a relative ratio of approx. 1 was observed in 7-month-old mice (Fig. 2d, Supplementary Fig. 2a–d), resembling the human 3R/4R tau protein ratio. Markedly increased 3R tau protein levels were observed in older mice (13 months) possessing extensive plaque pathology and in tau-KI alone (Fig. 2d), which argues against a contribution of amyloid in changing the 3R/4R tau ratio with age in our model. This difference was more pronounced in male mice of both, htau-KI and 5xFADxhtau-KI, genotypes compared to female mice (MANOVA with post hoc Bonferroni: females: *P*=5.1e^−22^; males: *P*=3.68e^−36^). Furthermore, distinct 3R and 4R tau isoforms could be visualized on Western blots of dephosphorylated brain extracts from htau-KI mice at the ages 3–12 months (Fig. 2e) suggesting expression of all 6 major tau isoforms. Histochemical analysis supported the notion that the 3R splice isoforms are not expressed in WT and 5xFAD mice, whereas both 3R and 4R isoforms are seen in htau-KI and 5xFADxhtau-KI mice (Fig. 2f). In summary, 4R tau is the only detectable isoform in mice possessing the mouse tau background (WT and 5xFAD). In mice with human tau-KI, an age- and sex-independent predominance of 3R tau over 4R tau is seen in htau-KI and 5xFADxhtau-KI mice at the mRNA level. At the protein level, this predominance of 3R has been detected in older, particularly male mice, whereas younger mice show a relative ratio of 3R/4R of approx. 1 suggesting a sex- and age-related impairment of tau homeostasis in this model.

### Correlating gene expression signatures across species reveals an interaction between amyloid and human tau

Next, we performed gene expression analysis using a specifically designed AD expression panel from NanoString [26] to assess the effects of high cerebral amyloidosis background (5xFAD) on our newly created htau-KI model. The comparison of WT, htau-KI, 5xFAD, and 5xFADxhtau-KI models revealed that a large fraction of variation in gene expression observed between WT and 5xFADxhtau-KI could be attributed to the 5xFAD model (Supplementary Fig. 1, Supplementary Tab. 1, 2).

**Fig. 3:**
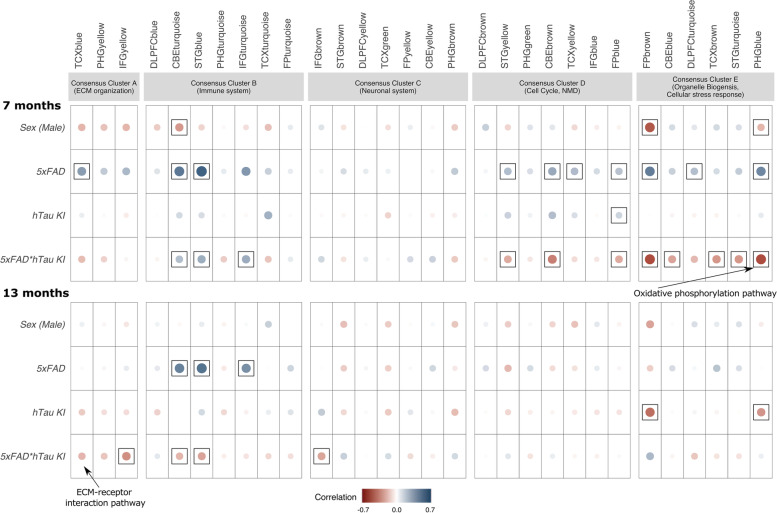
Correlation of gene expression data of studied mouse models with human data on post mortem brain regions reveals a seemingly protective effect of human tau on amyloid background in 5xFADxhtau-KI mice. Correlation analysis between mouse and human data was performed for 30 AMP-AD co-expression modules from seven brain regions and three clinical cohorts. Mouse effects for the 5xFAD and htau-KI models are relative to age-matched B6 female mice, which were correlated with the effects of human cases versus controls. In addition, an interaction term was introduced to correlate 5xFAD and htau-KI models with the effects of human cases compared to controls. Significant correlations (FDR-adjusted *P*<0.05) across species are highlighted by black rectangles. A strong positive correlation with immune related co-expression modules in consensus cluster B was observed for 5xFAD mice at 7 and 13 months. A strong negative correlation in modules enriched for genes in mitochondrial function (consensus cluster E) was shown for the htau-KI model solely on the 5xFAD background at the age of 7 months supporting an amyloid-dependent protective effect of htau. This includes modules enriched for specific disease pathways, such as the KEGG oxidative phosphorylation pathway in the PHGblue module from the MSSM cohort. At 13 months, this effect in the 5xFADxhtau-KI model was observed in human co-expression modules linked to extracellular matrix organization and immune function (consensus clusters A and B)

We then compared gene expression data from our mouse models to post mortem brain data from human AD patients using a previously described systems biology approach. This allowed us to assess the disease relevance of novel AD-associated mouse models [26]. The results from the multiple linear regression analysis revealed distinct effects between the 5xFAD and htau-KI mouse models when correlated to 30 human co-expression modules derived from seven brain regions and three independent cohorts (Fig. 3). These co-expression modules describe major sources of AD-related alterations across three post mortem brain cohorts from the AMP-AD consortium [38]. 5xFAD mice showed a moderate to strong positive correlation (correlation coefficients: 0.38–0.67, *P* < 0.01) with immune related co-expression modules from human post mortem brain samples in the cerebellum, superior temporal gyrus and the inferior frontal gyrus at 7 and 13 months of age (Fig. 3). This is in line with the results from the differential gene expression analysis and multiple recent studies highlighting an early activation of inflammatory pathways in 5xFAD mice [39, 40]. Furthermore, we observed weak, positive correlations (correlation coefficients < 0.1, *P* < 0.05) with multiple human co-expression modules in the 5xFAD mice at 7 months associated with neurodegenerative processes in consensus clusters C and D (Fig. 3). These include human modules from several brain regions and are annotated with multiple disease-specific pathways, such as the neuronal system, myelination, and neuronal development. Notably, gene expression correlation with human co-expression modules was weaker in the htau-KI genotype when compared to the 5xFAD mice (see also Additional file 2), indicating that the effect of human tau expression in htau-KI mice on pathways that are typically altered in AD is not as pronounced as in 5xFAD mice.

An interaction term was introduced in our linear mixed model to highlight positive or negative correlations between the 5xFAD and htau-KI models. Interestingly, we found a strong negative correlation between both genotypes associated with multiple human co-expression modules (Fig. 3, see also Additional file 3). This becomes apparent when comparing correlation coefficients between 5xFAD and 5xFADxhtau-KI. For example, a significant negative correlation (correlation coefficients −0.23 to −0.49, FDR-adjusted *P* < 0.01) between the 5xFADxhtau-KI and multiple human modules in consensus cluster E at 7 months of age was observed (Fig. 3), which is opposite to the positive correlation of the same modules in 5xFAD. These human modules that derive from the parahippocampal gyrus (PHGblue) and frontal pole brain regions (FPbrown) from the Mount Sinai brain bank cohort (MSBB) contain primarily genes implicated in oxidative phosphorylation and mitochondrial function. They are enriched for multiple cell-type-specific markers but not a single cell type. Furthermore, a negative correlation between 5xFADxhtau-KI and human co-expression modules linked to extracellular matrix organization and immune function was observed at 13 months of age (correlation coefficients: < −0.5, FDR-adjusted *P* < 0.01) (Fig. 3), which is again opposite to a positive correlation seen in 5xFAD. These modules are mainly enriched in astrocyte-specific genes and genes associated with signaling pathways implicated in extracellular matrix formation and energy metabolism. As can be deduced from the examples given above, the presence of htau in 5xFADxhtau-KI mice results in a negative correlation with human expression signatures, suggesting a seemingly protective effect on Aβ-induced pathology driven by the 5xFAD background. This applies to correlations in distinct consensus clusters enriched for genes associated with the immune system, organelle biosynthesis, and extracellular matrix organization. These effects are not evident in htau-KI mice. While our correlation analysis can assess significant overlaps in gene expression signatures across species, it is limited in identifying common pathways within the large co-expression modules. We performed gene set enrichment analysis (GSEA) [28] in order to identify significantly enriched pathways across species that are linked to the observed seemingly antagonistic effects of the 5xFAD- and 5xFADxhtau-KI backgrounds. GSEA aggregates the per gene statistics across multiple pathways, revealing even small but consistent transcriptional changes on the pathway level in a specific direction. At 7 months, multiple pathways in the 5xFADxhtau-KI model showed a significant enrichment when compared to the 5xFAD model (Supplementary Fig. 3a). These pathways are linked to AD-specific processes, including lysosomal function, oxidative phosphorylation, and phagocytosis. Among these pathways, genes in the KEGG oxidative phosphorylation (oxphos) pathway were coordinatively upregulated in the presence of htau-KI (5xFADxhtau-KI) when compared to the 5xFAD model (mmu190, enrichment score= 0.69, adjusted *P*-value= 0.0058) (Fig. 3, Supplementary Fig. 3a). In contrast, genes in the oxphos pathway (hsa190, enrichment score= −0.59, adjusted *P*-value= 0.0025) were downregulated in deceased AD patients from the MSBB cohort when compared to non-demented controls (Supplementary Fig. 3b–d) (see also: https://agora.ampadportal.org/genes for detailed human gene expression profiles). These findings in 7-month-old mice support the notion that the presence of htau on the 5xFAD background may prevent deficits in oxidative phosphorylation driven by amyloid pathology.

At 13 months of age, multiple pathways, which are linked to extracellular matrix and immune function, are downregulated in the 5xFADxhtau-KI model while the calcium and oxytocin signaling pathways are upregulated when compared to the 5xFAD model (Supplementary Fig. 4a). The ECM-receptor interaction is upregulated (hsa04512, enrichment score= 0.67, FDR-adjusted *P*-value= 0.0088) in the TCX brain region from the Mayo cohort associated with the TCXblue co-expression module (Fig. 3) and downregulated in the 5xFADxhtau-KI model (mmu04512, enrichment score= −0.72, FDR-adjusted *P*-value= 0.011) (Supplementary Fig. 4 a, b). Among the genes, which showed decreased expression in the 5xFADxhtau-KI model compared to the 5xFAD model, were multiple AD candidate genes (Supplementary Fig. 4c), which are significantly upregulated in AD patients compared to controls. *ITGB5* is an integrin that facilitates cell-extracellular matrix adhesion, which has been shown to act as a transcriptional hub gene for inflammatory AD modules [41]. *LAMB2* has been highlighted as an important hub gene in an integrated proteomic and transcriptomic study in the ROS/MAP cohort [42].

In summary, the expression of human tau on an amyloid background in 5xFADxhtau-KI mice alters transcriptional profiles of disease-associated pathways (mainly oxidative phosphorylation and mitochondrial function), which are found to be activated across post mortem brain regions of AD patients.

### Formation of pathological tau is linked to human tau expression

**Fig. 4:**
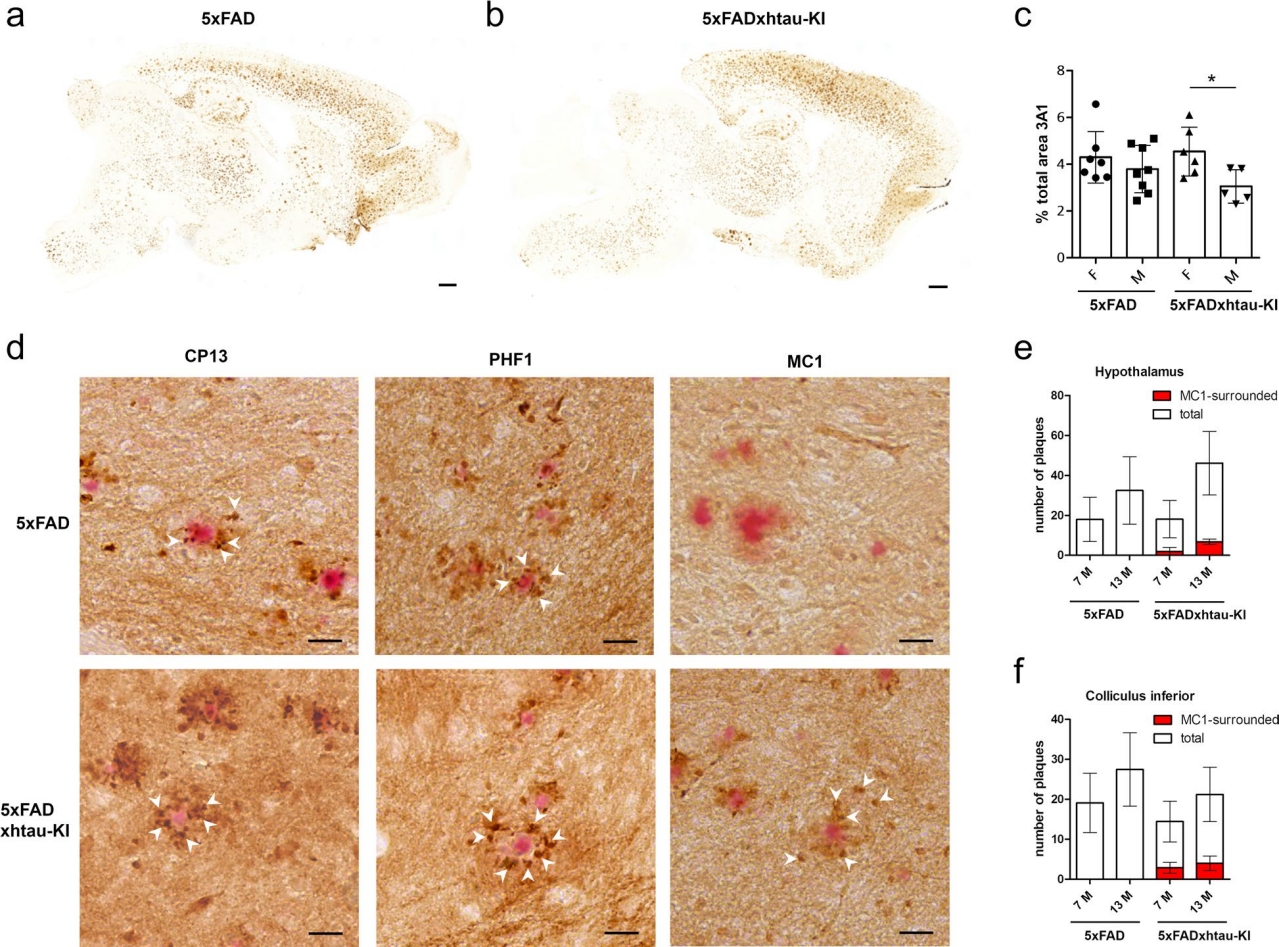
Analysis of tau and Aβ pathology. **a**,**b** Aβ staining using 3A1 antibody in representative sagittal brain slices of female, 13-month-old 5xFAD (**a**) and 5xFADxhtau-KI (**b**) mice. Scale 500 μm. **c** Quantification of the occupied total plaque area in brain slices analogous to **a** and **b** in female (F) and male (M) 13-month-old 5xFAD and 5xFADxhtau-KI mice. Plaque area in WT and htau-KI mice was equal to zero (not shown). Mean±SEM. *n*=5–8. * *P* < 0.05 unpaired *t*-test. **d** Sections from the hypothalamic region of 13-month-old, female 5xFAD and 5xFADxhtau-KI mice showing Congo red-stained Aβ plaques surrounded by dystrophic neurites (arrow heads). Three different antibodies were used for DAB staining of tau protein: CP13 (pathologic tau), PHF1 (later stage tangles), and MC1 (conformation-specific). Note that MC1-positive tau occurs exclusively in 5xFADxhtau-KI. Scale 20 μm. **e** Quantification of total and MC1-surrounded plaques in the hypothalamus of female 5xFAD and 5xFADxhtau-KI mice of 7 months (7 M) and 13 months (13 M) of age. Mean ± SD. 5xFAD, 7 M (*n*=9); 5xFAD, 13 M (*n*=6); 5xFADxhtau-KI, 7 M (*n*=7); 5xFADxhtau-KI, 7 M (*n*=6). **f** Quantification of total and MC1-surrounded plaques in the inferior colliculus of female 5xFAD and 5xFADxhtau-KI mice of 7 months (7 M) and 13 months (13 M) of age. Mean ± SD. 5xFAD, 7 M (*n*=8); 5xFAD, 13 M (*n*=5); 5xFADxhtau-KI, 7 M (*n*=7); 5xFADxhtau-KI, 7 M (*n*=6)

Given the observed strong hints for a negative correlation between cerebral amyloidosis and human tau, we were interested in possible effects on brain histopathology. First, Aβ staining using antibody 3A1 was analyzed in all four genotypes. Figure 4a and b depict representative sagittal brain slices of female, 13-month-old 5xFAD and 5xFADxhtau-KI mice. Quantification of overall plaque deposition revealed no statistically significant differences in the total plaque area between 5xFAD and 5xFADxhtau-KI at 7 and 13 months of age (Fig. 4c, Supplementary Fig. 5b-f). No Aβ-positive staining was found in WT and htau-KI mice (data not shown). In addition, the inflammatory phenotype expected for 5xFAD mice could also be seen in 5xFADxhtau-KI crossbreds, but not in htau-KI, as studied by astroglial (GFAP, Supplementary Fig. 6a, c) and microglial (Iba-1, Supplementary Fig. 6b, d) staining.

Three different tau-antibodies have been applied to visualize pathological tau forms in brain slices. PHF1-positive (phospho-Ser396/Ser404) and CP13-positive (phospho-Ser202) dystrophic neurites were found in close proximity of plaque depositions in all studied mice of the 5xFAD and 5xFADxhtau-KI genotype, independent of age (7 and 13 months) and sex. Figure 4d shows representative PHF1 and CP13 stainings of the hypothalamic region of 13-month-old female 5xFADxhtau-KI mice. Note that these stainings do not reflect simple adsorption of the antibody at plaques but instead indicate Aβ-dependent neuritic pathology as also seen in other AD mouse models [43, 44]. Neither PHF1 nor CP13 immunoreactivity was observed in WT and htau-KI mice (not shown).

The antibody MC1 targets a disease-specific conformational modification of tau because binding occurs to both the N-terminus (aa 7-9) and an epitope in the third microtubule binding domain (aa 313-322) [45, 46]. In contrast to PHF1 and CP13, only 5xFADxhtau-KI, but not 5xFAD, show MC1-positive stainings of dystrophic neurites near plaques (Fig. 4d) in different brain regions, such as cortex, hippocampus, thalamus, hypothalamus, and inferior colliculus, independent of sex and age. Highest number of plaque-associated MC1-positive dystrophic neurites was observed in hypothalamus and inferior colliculus. Therefore, quantification was done in these two small and well-defined areas, which are also affected in human AD. In the hypothalamus (Fig. 4e) and inferior colliculus (Fig. 4f) of female 5xFADxhtau-KI mice, MC-positive dystrophic neurites were found 10–20 % of total plaques (hypothalamus: 7 M: 10.3 %; 13 M: 15,3 %; colliculus inferior: 7 M: 19.8 %; 13 M: 18,8 %). In summary, different brain regions of 5xFADxhtau-KI mice possess signs of early changes in pathological tau formation that were independent of the PHF1 and CP13 markers. Highest MC1-immunoreactivity occurred in the vicinity of plaques and seemed to be enhanced by amyloid deposition.

### Electrophysiological measurements reveal genotype-specific deficits in short-term plasticity, LTP, and elementary synaptic function

The transcriptomic, biochemical, and histopathological analyses presented above strongly indicated that in 5xFADxhtau-KI mice multiple interactions occur between the 5xFAD and the htau background, and that these interactions are apparently correlated to certain functional circuits (modules) in post mortem tissue from human AD patients. The question was, however, whether these statistical interactions and correlations result in detectable phenotypes in functional parameters at the cellular and behavioral level. Given that AD is a synaptopathy [47], we first applied electrophysiological methods to measure parameters of basal synaptic function and synaptic plasticity in the CA1 region of the hippocampus in vitro. The hippocampus is one of the regions that is affected early by Aβ pathology in humans, subsequently to the initial infiltration of the neocortex [48]. Its CA1 region is particularly vulnerable to any synaptotoxic effects [49] and was therefore selected as region of interest.

**Fig. 5:**
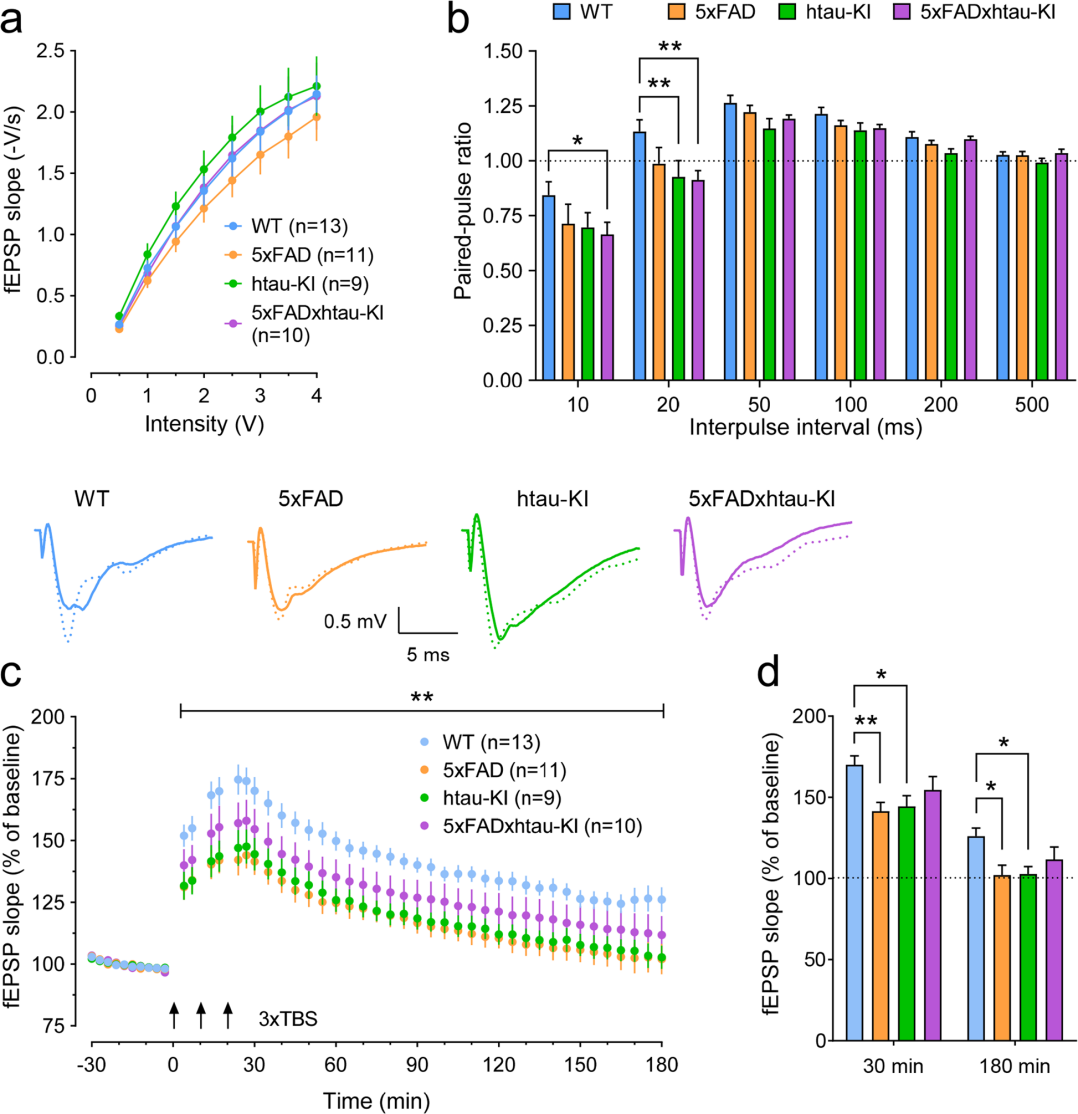
Characterization of basal synaptic transmission, paired-pulse responses, and long-term potentiation in the hippocampal CA1-region of about 8-month-old 5xFAD, htau-KI, and 5xFADxhtau-KI mice compared to WT animals. **a** Basal synaptic transmission was similar across all genotypes. **b** Paired-pulse stimulation led to lower ratios in htau-KI and 5xFADxhtau-KI mice, but only at the shortest interpulse intervals (10–20ms). **c** LTP induced by 3× theta-burst stimulation (TBS) was impaired in htau-KI and 5xFAD mice compared to WT controls (RM-ANOVA + Dunnett’s multiple comparison tests). The asterisks above the line on top of the LTP time series indicate the significant main effect of genotype. Insets show representative signal traces for every genotype during baseline (solid line) and at 180 min (dotted line). **d** Bar graphs exemplify the magnitudes of potentiation for each genotype for LTP induction (at 30 min post-TBS) and maintenance (at 180 min). Tukey’s multiple comparisons tests confirmed significant differences for htau-KI and 5xFAD. The combined 5xFADxhtau-KI model expresses an LTP of an intermediate magnitude that is not significantly different from WT mice. Numbers between brackets indicate sample sizes (animals). Mean ± SEM is given, *P < 0.05, **P < 0.01. For all: WT (*n*=13), 5xFAD (*n*=11), htau-KI (*n*=9), 5xFADxhtau-KI (*n*=10)

As shown in Fig. 5a, recordings of field excitatory post-synaptic potentials (fEPSPs) in about 8-month-old animals (see “Methods” for details) showed no genotype differences in basal synaptic transmission as evaluated by input/output curves. In contrast, paired-pulse ratios as a measure of presynaptically mediated short-term plasticity were significantly lower in htau-KI and 5xFADxhtau-KI mice at the lowest interpulse intervals (10–20 ms, Fig. 5b). This is indicative of altered GABA_A_ receptor-mediated inhibition. When long-term potentiation (LTP), the most established cellular model for memory formation and storage [50], was examined, we found a significant overall genotype effect (F_3,39_ = 5.264, *P* = 0.0038; Fig. 5c). Post hoc multiple comparisons of the potentiation at 30 and 180 min (Tukey test) detected significant impairments in LTP induction and LTP maintenance, respectively, in 5xFAD and htau-KI mice (Fig. 5d). Compared to the latter two genotypes, LTP in 5xFADxhtau-KI mice was less compromised and not statistically different from WT controls.

**Fig. 6:**
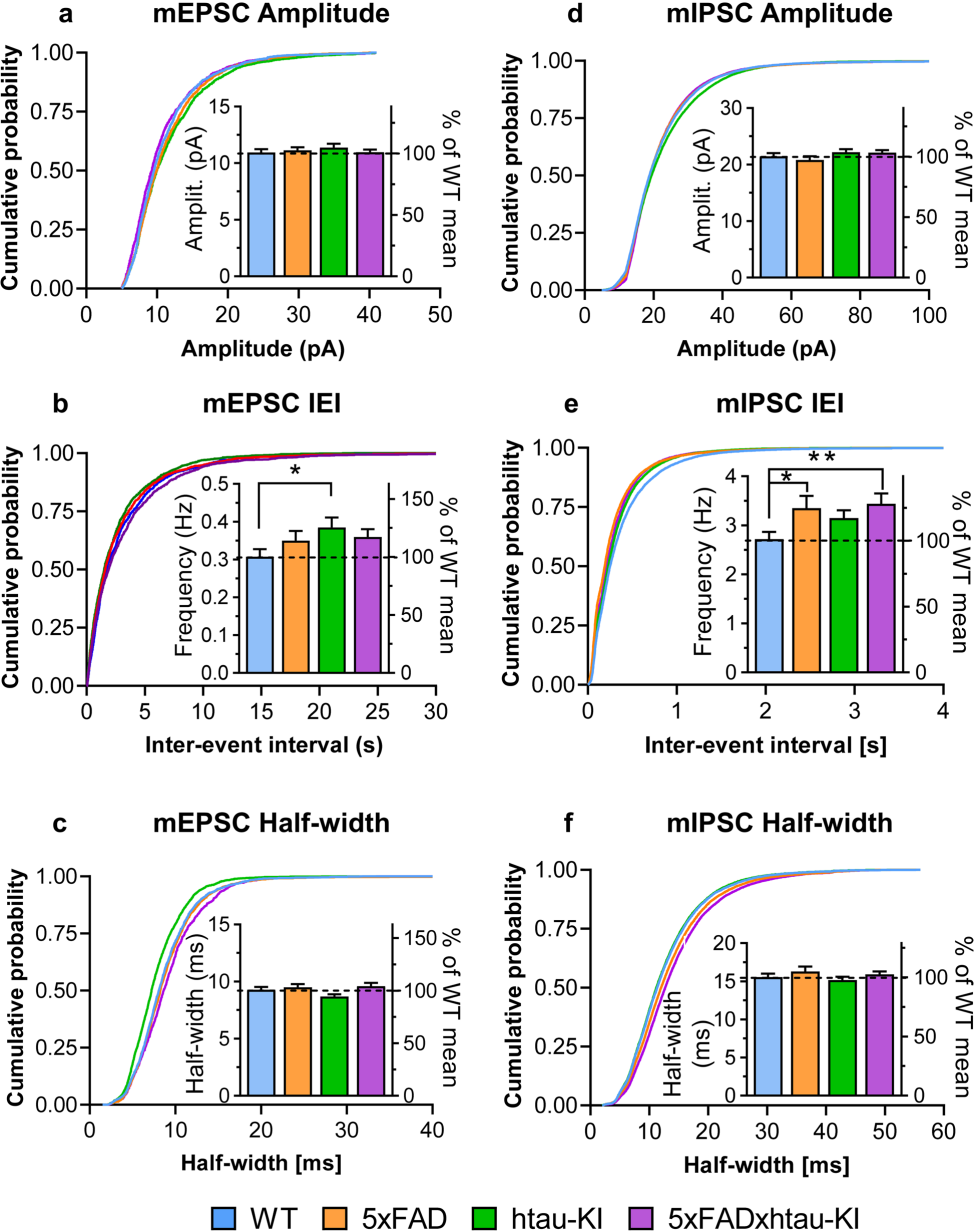
Analysis of action potential–independent mEPSCs and mIPSCs reveals enhanced frequencies in about 8-month-old 5xFAD, htau-KI mice, and 5xFADxhtau-KI. **a** Mean amplitude of mEPSCs is unchanged in the three genetically modified mouse strains as compared to WT (See insets in **a**). Mean ± SEM. WT (*n*=41), 5xFAD (*n*=38), htau-KI (*n*=43), 5xFADxhtau-KI (*n*=38). **b** Mean frequency of mEPSCs of htau-KI mice is significantly increased compared to WT controls, but the increased means in 5xFAD and 5xFADxhtau-KI mice fail to reach statistical significance (See insets in **b**). Mean ± SEM. WT (*n*=41), 5xFAD (*n*=38), htau-KI (*n*=43), 5xFADxhtau-KI (*n*=38). **c** mEPSC half-width, a measure that characterizes the kinetics of inactivation does not differ across genotypes. Analyses of the probability distributions of the data (**a**–**c**) revealed significant genotype differences for all three parameters. Mean ± SEM. WT (*n*=40), 5xFAD (*n*=37), htau-KI (*n*=42), 5xFADxhtau-KI (*n*=37). **d** Similar to the mean mEPSC amplitudes (inset in a), the mean mIPSC amplitudes do not differ between genotypes (inset in **d**). Mean ± SEM. WT (*n*=42), 5xFAD (*n*=38), htau-KI (*n*=43), 5xFADxhtau-KI (*n*=38). **e** Frequency of mIPSCs in htau-KI and 5xFADxhtau-KI mice is markedly increased (inset in **e**). Mean ± SEM. WT (*n*=42), 5xFAD (*n*=38), htau-KI (*n*=43), 5xFADxhtau-KI (*n*=38). **f** Kinetics of mIPSC inactivation as measured by the half-width was very similar in all genotypes (inset in **f**). Kolmogorov-Smirnov tests yielded significant genotype differences for the amplitude, the frequency, and the half-width of mIPSCs (**d**–**f**). Mean ± SEM. WT (*n*=42), 5xFAD (*n*=38), htau-KI (*n*=43), 5xFADxhtau-KI (*n*=38). Statistics: Welch’s one-way ANOVA test using the two-stage linear step-up procedure of Benjamini, Krieger, and Yekutieli. *P < 0.05, **P < 0.01. Cumulative distribution functions of mEPSCs and mIPSCs were compared by Kolmogorov-Smirnov test

To check whether the genetically modified mouse lines show any changes at the level of elementary synaptic function, we used whole-cell patch-clamp recordings of mEPSCs and mIPSCs from CA1 pyramidal neurons (Fig. 6). Striking differences between the genotypes were detected in the miniature current frequencies, especially in the frequency of the mIPSCs resulting in a significant effect of genotype (Fig. 6e; W_(3, 84.3)_ = 3.362; *P* = 0.0225, Welch’s one-way ANOVA). Post hoc tests confirmed enhanced mIPSC frequencies of 5xFAD and 5xFADxhtau-KI mice compared with WT (5xFAD: *P* = 0.0337; 5xFADxhtau-KI *P* = 0.0066, Dunnett-T3 post hoc tests). The increase in mEPSC frequencies as apparent in the inset in Fig. 6b was however less pronounced resulting only in a significant increase of htau-KI mEPSCs (*P* = 0.0231, Dunnett-T3 post hoc test). Interestingly, no differences could be detected in the mean amplitudes of mEPSCs and mIPSCs across all genotypes (insets in Fig. 6a, d). Likewise, mEPSC and mIPSC half-width, an indicator of charge transfer and kinetics, did not differ across genotypes (insets in Fig. 6c, f). Further analysis of the mEPSCs and mIPSCs by comparing the probability distributions of the data with the Kolmogorov-Smirnov test confirmed genotype differences for those parameters that had significantly different mean values as mentioned above. For example, the cumulative probability plots of the inter-event intervals (IEIs; the reciprocal of the frequency) of mIPSCs of transgenic mice (Fig. 6e) showed a significant shift to the left compared to WT, towards shorter IEIs (i.e., higher frequencies). However, the analysis also detected significant genotype differences in the cumulative probability distribution of several other parameters (mIPSC amplitude, mIPSC inter-event interval, mIPSC half-with: all *P* < 0.0001, mEPSC amplitude: WT vs. htau-KI *P* < 0.0001, WT vs. 5xFADxhtau-KI *P* = 0.0061, mEPSC inter-event interval: WT vs. 5xFAD *P* = 0.0005, WT vs. htau-KI *P* < 0.0001; mEPSC half-width: WT vs. htau-KI *P* < 0.0001, WT vs. 5xFADxhtau-KI *P* < 0.0001, not shown).

#### Behavioral analysis of 5xFADxhtau-KI mice in comparison to htau-KI, 5xFAD, and WT littermates

**Fig. 7:**
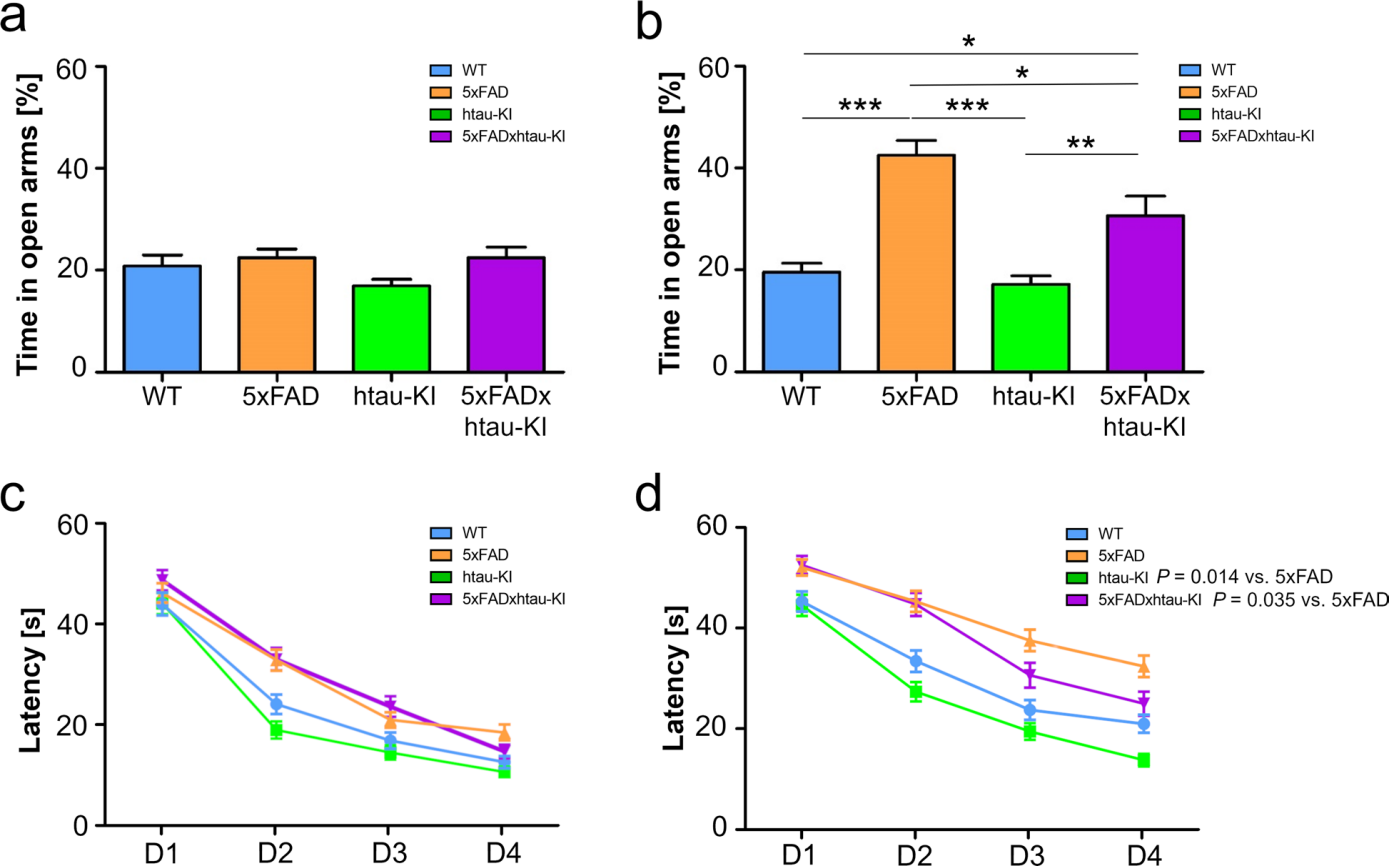
Decreased anxiety and impaired spatial learning in 12-month-old 5xFAD and 5xFADxhtau-KI mice compared to WT and htau-KI mice. **a**,**b** Results of the elevated plus maze do not show significant differences at the age of 6 months (**a**), but at the age of 12 months, the time spent on open arms in 5xFAD and 5xFADxhtau-KI mice is significantly different compared to WT and htau-KI (**b**). Mean ± SEM. One-way ANOVA with Tukey’s post hoc test, *P < 0.05, **P < 0.01, ***P < 0.001, 6 months: WT (*n*=23), 5xFAD (*n*=23), htau-KI (*n*=20), 5xFADxhtau-KI (*n*=20) (male and female); 12 months: WT (*n*=22), 5xFAD (*n*=21), htau-KI (*n*=23), 5xFADxhtau-KI (*n*=18) (male and female). **c**,**d** Latency to find a hidden platform in the Morris water maze analyzed in 6-month-old (**c**) and 12-month-old (**d**) animals. While there was no significant interaction of genotype and trial days on latency in 6-month-old mice (all *P*-values >0.05), we found a significant interaction of genotype and trial on slope of latency curves of 5xFAD mice compared to 5xFADxhtau-KI mice and htau-KI mice, respectively (5xFAD vs. 5xFADxhtau-KI: *P*=.0.035; 5xFAD vs. htau-KI: *P*= 0.014). Each data point represents the mean ± SEM of 4 trials per day. *P*-values from RM-ANOVA using slopes of learning curves are depicted in the Fig. 6 months: WT (*n*=23), 5xFAD (*n*=24), htau-KI (*n*=21), 5xFADxhtau-KI (*n*=20) (male and female); 12 months: WT (*n*=24), 5xFAD (*n*=23), htau-KI (*n*=24), 5xFADxhtau-KI (*n*=19) (male and female)

Given the phenotypes associated with different synaptic measures, we addressed the question to what extent these synaptic phenotypes are paralleled by changes in behavior. Therefore, we analyzed mice of all study groups at the age of 6 and 12 months. At the age of 6 months, no differences among groups were found in exploratory and anxiety-like behavior in the elevated plus maze (EPM) (Fig. 7a, b). At the age of 12 months, there was a significant effect of genotype on the time spent in open arms. Both 5xFAD and 5xFADxhtau-KI mice explored open arms longer than WT and htau-KI mice. In addition, there was also a significant difference between 5xFAD and 5xFADxhtau-KI, with 5xFADxhtau-KI spending less time in the open arms corroborating findings from electrophysiology and suggesting a partial rescue of the phenotype.

Lastly, we tested hippocampus-dependent spatial learning with the hidden platform version of the Morris water maze. Figure 7c, d shows the mean latency time to reach the platform for each genotype each day and their standard errors of the mean for 6-month-old mice (Fig 7c) and 12-month-old mice (Fig. 7d), respectively. The differences between the slopes of learning curves of the four genotypes were assessed by conducting a RM-ANOVA using the lmerTest package in R. There was no evidence for an effect of the interaction of genotype and trial days on latency in 6-month-old mice (all *P*-values >0.05). In contrast, we found a significant effect of the interaction of genotype and trial on slope of latency curves of 5xFAD mice compared to 5xFADxhtau-KI mice and htau-KI mice, respectively (5xFAD vs. 5xFADxhtau-KI: *P*=0.035; 5xFAD vs. htau-KI: *P*=0.014). Additionally, we analyzed the distance covered by an animal to reach the platform (Supplementary Fig. 7a, b) and swim speed (Supplementary Fig. 7c, d) as score for cognitive learning curves as they also reflect the actual spatial learning abilities.

In conclusion, our behavioral experiments on 6-month-old mice do not show any significant difference between 5xFAD mice and either one of the listed genotypes. Experiments on 12-month-old mice revealed a statistically significant difference in exploratory and anxiety-like behavior tested in EPM. In addition, better spatial learning abilities were observed in 5xFADxhtau-KI and htau-KI compared to 5xFAD.

## Discussion

Here, we have presented novel findings on correlations of htau-KI expression under progressing cerebral amyloidosis in 5xFAD mice. Several studies suggested Aβ as initiating factor of tau toxicity [9, 11, 51]. A closer look into our mouse lines by gene expression analysis, immunohistochemical staining, and electrophysiology suggested differential effects of human tau and murine tau.

We observed a positive correlation with multiple immune-associated co-expression modules from post mortem human brain samples in comparison to the 5xFAD model. This is in line with the differential expression analysis across strains, highlighting an increased expression of immunological genes in 5xFAD mice linked to the activation of microglia and astrocytes. In contrast to the 5xFAD model, htau-KI mice showed no significant differences in the levels of differentially expressed genes when compared to WT mice. In addition, the correlations with human gene expression modules suggest a subtle effect of the htau-KI allele that is absent in the 5xFAD model. Interestingly, this effect of htau-KI on gene expression is more pronounced in the presence of amyloid in the 5xFADxhtau-KI mice. This seemingly antagonistic effect between amyloid and the expression of human tau in a murine background was associated with a strong negative correlation of co-expression modules from deceased AD patients. We identified multiple genes that showed small but consistent transcriptional changes on the co-expression module and pathway level, which are enriched in 5xFADxhtau-KI mice and linked to mitochondrial function, energy metabolism, and extracellular matrix function. These genes show the opposite effect in terms of gene expression when compared to deceased AD patients. An explanation could be an insufficient activation of AD-related signaling cascades and their crosstalk by human instead of murine tau in murine background.

In 5xFADxhtau-KI mice at 7 months of age, multiple genes in the oxphos pathway are increased, while they are downregulated in AD patients compared to cognitively normal elderly controls. Mitochondria dysfunction is a prominent early feature in the pathogenesis of AD, which has been linked to tau phosphorylation and aggregation in AD patients and multiple animal models [52]. Interestingly, Trease et al. examined synaptic and non-synaptic brain mitochondria of 5- and 8-month-old htau mice, which express wild-type human tau in all 6 isoforms in the absence of murine tau [53]. Using subcellular proteomics, bioenergetics assessment, and metabolic pathway analysis, the authors revealed alterations in multiple pathways with oxidative phosphorylation yielding the strongest negative activation score for synaptic mitochondria of 8-month-old mice. Pathologic forms of tau were found to associate preferentially with synaptic mitochondria, and the maximal respiration of htau synaptosomes was impaired. Thus, apparently, the accumulation of non-mutant human tau at the synapse may compromise proper mitochondrial operation with detrimental consequences for synaptic functioning [53]. We also observed a decreased expression of genes implicated in extracellular matrix and immune functions in our 5xFADxhtau-KI model at an older age. These genes showed an increased expression in AD patients, supporting the idea that the observed effect of htau might protect cells from oxidative damage at an early stage, which could in turn promote ECM cross-linking and immune function at a later disease stage.

Furthermore, a molecular response of tau in the vicinity of plaques by formation of neuritic pathology was observed in 5xFAD and 5xFADxhtau-KI mice, consistent with previous reports from 5xFAD mice and other AD mouse models carrying human mutations in APP and PS1 [54, 55]. Here, amyloid-expressing models shared phosphorylation sites stained with PHF1 and CP13 antibodies. However, phosphorylation at distinct amino acids alone does not allow discrimination of normal tau from pathological tau forms [56, 57]. Noteworthy, the conformation-dependent MC1 epitope was identified in dystrophic neurites in the vicinity of plaques of 5xFADxhtau-KI mice of both tested ages and sexes. This is of importance since MC1-positive changes are among the earliest changes in AD brains preceding PHF [58]. Aragão Gomes and colleagues have recently shown that MC1-positive changes further indicate maturation of tau aggregates from diffusely distributed T231-positive and S396/pS404-positive tau in the somatodendritic compartment and white matter/neuropil, respectively to pretangles [57]. There, MC1 positivity was found to be a last step before Gallyas-positive NFTs appeared. Obviously, these changes require the presence of human tau in our model. Since the C-terminal epitope for MC1 is identical in murine and human tau, the exclusive staining of MC1-positive material in htau-KI and 5xFADxhtau-KI mice could be due to (I) a missing specificity of MC1 for murine tau, caused by the amino acid substitution E_9_D in murine tau or (II) a lack of pathologically folded and therefore MC1-positive murine tau in 5xFAD and WT mice. The preservation of MC1-binding in murine tau is supported by several studies and suggests that murine tau could adopt a folding state that is detected by MC1 [59, 60]. Therefore, despite high similarity in primary structure and phosphorylation pattern between human and murine tau [43, 44], murine tau lacks intrinsic properties in order to misfold in similar manner as human tau, as observed in the present study. This difference is further supported by the above discussed gene expression analysis.

Furthermore, human-like tau splicing into 6 isoforms is observed in htau-KI and 5xFADxhtau-KI mice, with endogenous levels of protein expression in younger mice. Quantification of the mRNA levels of 3R and 4R tau isoforms revealed a substantial shift from 4R to 3R tau in these mice, leading to an approx. 20-fold higher expression of 3R tau independent of sex and age. However, at the protein level, only aged mice show the 4R to 3R shift with a 4-fold (female) to 14-fold (male) higher protein load of 3R tau, with younger mice possessing the expected ratio of 3R/4R tau at approx. 1. This is interesting since mice normally do not express 3R tau [16, 17] and artificially supplied 3R tau via the knock-in may not be well degraded and may accumulate with increasing age. Furthermore, it has been shown that 3R tau is able to influence the assembly of 4R tau on the protein level in vitro, suggesting a fine balance between both isoforms [61]. This balance could be lost in our model upon aging. In addition, a substantially higher 3R expression was also shown by Andorfer and colleagues in their MAPT mouse model [62]. Saito et al. recently found equal molar ratios of 3R and 4R in their *MAPT* KI mice by semi-quantitative PCR; however, the authors did not report a higher amount of 0N3R vs. 0N4R tau protein [7]. Therefore, we hypothesize that mice with humanized tau expression show a higher expression of 3R vs. 4R tau and that differences in mRNA are probably not readily detected when using semi-quantitative RT-PCR to quantify the different tau isoforms. With respect to humans, an equal ratio of 3R/4R tau is described for both healthy individuals and AD patients [63]. Hence, our current model is limited in modeling human-like ratios of tau isoforms in aged mice. Another group has successfully tried to balance the ratio between 3R and 4R tau in their 6hTau mouse model [64].

The histopathological, biochemical, and molecular analyses point to a role of human tau in multiple interactive mechanisms between the 5xFAD and the htau background in 5xFADxhtau-KI mice. However, the relevance of such interactions is best demonstrated by detectable phenotypes in functional parameters at the cellular and behavioral level. In this regard, we found a significant impairment of long-term potentiation (LTP), a form of synaptic plasticity and candidate mechanism for memory formation and storage at the cellular level, in 5xFAD and htau-KI mice. Although there seems to be an LTP deficit in 5xFADxhtau-KI mice, it did not reach the level of statistical significance. The LTP deficit in 5xFAD mice was expected and is in accordance to previous reports [65, 66]. The impaired potentiation in htau-KI mice, however, solely caused by the replacement of WT mouse tau with WT human tau, was somewhat unexpected. Due to the scarcity of studies with humanized WT-tau mouse models, this phenotype is difficult to explain. As mentioned above, Saito et al. generated a closely related humanized tau mouse line [7], but the authors have not yet reported on synaptic function of their *MAPT* KI mice. Therefore, the only other humanized WT mouse model to compare our data with, is the hTau mouse line, generated by crossbreeding of the 8c mice [67] and tau knock-out mice [68]. Polydoro et al. examined basal synaptic transmission, short-term plasticity, and LTP in hTau mice [69]. Twelve-month-old but not 4-month-old htau mice showed impaired LTP in response to high-frequency stimulation, thereby corroborating our finding that the replacement of murine by human tau may result in synaptic dysfunctions [69]. Such LTP deficit in htau mice could be due to differences in sequence and isoform expression between murine and human tau that may preclude interactions with murine pathways that are essential for LTP. However, the latter does not explain why in the study of Polydoro et al., 12-month-old hTau mice presented a normal LTP [69], induced by a similar theta-burst protocol as used in our study. The reason for this could be the different way tau humanization was achieved in the two mouse lines. In hTau mice, human tau is expressed via a human transgene, while a targeted whole-gene replacement was performed in our model. As a consequence, the regulation of human tau and its integration in the murine neural environment is likely to differ between hTau and htau-KI mice, resulting in different or missing LTP phenotypes. Furthermore, basal synaptic transmission in the CA1-region of our 8-month-old htau-KI mice did not differ between genotypes, resembling the unchanged input/output properties of Schaffer collateral/CA1 pyramidal cell synapses in 4-month-old hTau mice. Twelve-month-old hTau mice, in contrast, displayed an increased basal excitability, a very rare finding in AD and FTD mouse models according to our experience and the literature [70].

In order to better understand at an elementary level, the contribution of different genotypes to synaptic dysfunction via changes in pre- vs. post-synaptic processes, we measured action potential–independent mEPSCs and mIPSCs. The obtained significant increase in the mean frequency of mEPSCs in htau-KI mice, together with an unchanged mean amplitude and half-width, suggests an increased presynaptic activity of glutamatergic synapses in these mice due to the complete replacement of mouse tau by human tau. This effect of human tau still seemed to be present in the crossbred 5xFADxhtau-KI mice but reached only borderline significance. Strikingly, in 5xFAD mice, the combination of 5 different AD-promoting transgenes had apparently no effect on presynaptic glutamatergic function. Previous measurements of mEPSCs in other hippocampal and in cortical regions of 5xFAD mice led to variable results. Muller et al. found in the dentate gyrus of 12-month-old female 5xFAD mice a lower mEPSC frequency compared to WT mice [71] and Buskila et al. reported for 8–12-week-old 5xFAD mice decreased mEPSC amplitudes and frequencies of layer 5 pyramidal neurons in the somatosensory cortex, indicative of impaired pre- and post-synaptic function [72].

For mIPSCs, significant differences to WT littermates were detected in mIPSC frequencies of 5xFAD and 5xFADxhtau-KI mice. Given the very similar increase in frequency in the two strains, the 5xFAD background is likely to be responsible for the enhanced values in the crossbred strain. This is in contrast to the findings for mEPSCs, where the increase in htau-KI was the highest and the only one that reached significance. There are, to the best of our knowledge, no published mIPSC measurements in 5xFAD mice. Therefore, our data can only be compared to published values of other AD mouse models. Inspection of mIPSCs in hAPPJ20 mice expressing the APP Swedish and Indiana mutation [73] yielded increased frequencies compared to controls, whereas mIPSC amplitudes were unchanged [10]. The frequency of mEPSCs was reduced in hAPPJ20 mice, but interestingly, reduction of tau in these mice normalized the frequency of both mIPSCs and mEPSCs to control level. This suggests that tau is involved in the regulation of presynaptic function, which is supported by the effect of htau on glutamatergic and GABAergic synaptic function in htau-KI and 5xFADxhtau-KI mice in our study.

On the behavioral level, we found a significant effect of the interaction of genotype and trial on slope of latency curves of 5xFAD mice compared to 5xFADxhtau-KI mice and htau-KI mice. Whether the latency on 5xFADxhtau-KI mice compared to 5xFAD mice reflects a combined effect of 5xFAD in htau-KI background or a partial rescue by incomplete integration of htau into the murine background is currently unknown. It has been published that genetic ablation of murine tau rescues the effect on latency in amyloid-expressing mice [51]. Therefore, the reduced latency found in our study could be interpreted in several ways, combining effect or partial network integration.

Taken together, our data indicate that in our novel 5xFADxhtau-KI mice progressive amyloid and tau act and partly interact in convergent pathways. The detailed mechanisms leading to phenotypes that are seemingly consistent with an antagonistic effect of Aβ and tau in a cellular environment of increased amyloidosis cannot yet be disentangled with the current first data set. The same applies to the proposition of a protective role of human tau in certain processes under these conditions. Alternative explanations for the phenotypes observed include (i) the absence of murine tau, (ii) multiple effects of the expression of human tau in a murine environment, such as interactions of htau with crucial murine AD-related pathways that are different from interactions of murine tau, or the lack of a proper interaction of htau with these pathways, i.e., a loss of function, (iii) an expression pattern of the different htau isoforms that deviates in time and quantity from the intrinsic profile of murine tau. Our findings have implications for model generation in AD because they highlight the complex nature of Aβ/tau interactions in advanced AD mouse models. Our 5xFADxhtau-KI mice may not represent an improved model for AD, but are expected to be valuable for further studying interactions between human tau and amyloid in vivo.

### Limitations

The present study includes early stage pilot experiments. Furthermore, mouse models for Alzheimer’s disease are limited by the finding that this species does not develop the sporadic form of AD, which represents the vast majority of all AD cases. Therefore, findings from AD mouse models help to understand basic biological principles; however, their translation into human biology has been shown to be complex. Lastly, external proof of our results by repetition in different institutions would further strengthen the presented findings. However, by adherence to the 3R principles of Russell and Burch, the number of studied animals was limited to the absolutely required number in order to obtain statistically significant data.

## Conclusions

In conclusion, we have characterized a novel human htau-KI mouse model under progressing cerebral amyloidosis. We discovered that the interaction of native human tau with an increasing amyloid load shows unexpected differences at the transcriptional level compared to models expressing murine tau. This effect was not observed for the murine tau background despite its high structural similarity to human tau. Furthermore, the expression of human tau during progressing cerebral amyloidosis in 5xFAD mice changed transcriptional profiles of disease-associated pathways, which are found to be activated across post mortem brain regions of AD patients. In addition, we detected the presence of a confirmation-specific epitope (MC1) in dystrophic neurites in the vicinity of plaques. This finding required the htau-KI background, suggesting again important differential effects of murine and human tau. The results of our study might have implications for the design of next-generation AD mouse models. In summary, we suggest that cerebral amyloidosis in mice should be analyzed on the background of native human tau instead of murine tau expression.

## Supplementary Information

**Additional file 1: Supplementary Tab. 1.** Differential gene expression in WT vs. 5xFAD at an age of 13 months. **Supplementary Tab. 2.** Differential gene expression in WT vs. 5xFADxhtau-KI at an age of 13 months. **Supplementary Tab. 3.** Applied antibodies. **Supplementary Tab. 4.** Applied Primers for DNA amplification and 3R/4R cDNA sequences. **Supplementary Fig. 1.** Differential gene expression analyzed using nCounter Mouse AD gene expression panel. **Supplementary Fig. 2.** 3R and 4R tau expression in dephosphorylated brain extracts analyzed by Western Blot. **Supplementary Fig. 3.** Gene Set Enrichment Analysis in 5xFADxhtau-KI mice at 7 months of age. **Supplementary Fig. 4.** Gene Set Enrichment Analysis in 5xFADxhtau-KI mice at 13 months of age. **Supplementary Fig. 5.** Plaque quantification in 7 and 13 months old 5xFAD and 5xFADxhtau-KI mice. **Supplementary Fig. 6.** Analysis of inflammatory phenotype in 5xFAD and 5xFADxhtau-KI. **Supplementary Fig. 7.** Comparison of distance and swim speed among genotypes.

**Additional file 2.** Gene set enrichment analyses results.

**Additional file 3.** Correlation coefficients.

**Additional file 4.** Original image file Figure 1b_GAPDH.

**Additional file 5.** Original image file Figure 1b_Human tau.

**Additional file 6.** Original image file Figure 1b_Mouse tau.

**Additional file 7.** Original image file Figure 1d.

**Additional file 8.** Original image file Figure 1e_GAPDH_htau-KI.

**Additional file 9.** Original image file Figure 1e_GAPDH_WT.

**Additional file 10.** Original image file Figure 1e_total tau_htau-KI.

**Additional file 11.** Original image file Figure 1e_total tau_WT.

**Additional file 12.** Original image file Figure 2e_3R.

**Additional file 13.** Original image file Figure 2e_4R.

**Additional file 14.** Original image file Figure 2e_GAPDH.

## Data Availability

Additional information and material are available from the corresponding author on reasonable request.

## Abbreviations

AD: Alzheimer’s disease
AMP-AD: Accelerating medicines partnership program for Alzheimer’s disease
APP: Amyloid precursor protein
ECM: Extracellular matrix
EPM: Elevated plus maze
EPSC: Excitatory post-synaptic current
FAD: Familial Alzheimer’s disease
fEPSP: Field excitatory post-synaptic potentials
FP: Frontal pole
GABA: Gamma-aminobutyric acid
GSEA: Gene set enrichment analysis
IPSC: Inhibitory post-synaptic current
KEGG: Kyoto Encyclopedia of Genes and Genomes
KI: Knock-in
LTP: Long-term potentiation
MAPT: Microtubule-associated protein tau
MSBB: Mount Sinai brain bank
PHF: Paired helical filament
PHG: Parahippocampal gyrus
PS1: Presenilin 1
ROSMAP: Religious Orders Study and Memory and Aging Project
SHIRPA: SmithKline Beecham, Harwell, Imperial College, Royal London Hospital, phenotype assessment
TCX: Temporal cortex
WT: Wild type
