# Supplementary material for: A novel human tau knock-in mouse model reveals interaction of Abeta and human tau under progressing cerebral amyloidosis in 5xFAD mice
Source: Alzheimers Res Ther. 2023 Jan 14;15:16. doi: 10.1186/s13195-022-01144-y (PMC9840277; doi:10.1186/s13195-022-01144-y)
Supplement: Supplementary file 1 — Additional file 1: Supplementary Tab. 1. Differential gene expression in WT vs. 5xFAD at an age of 13 months. Supplementary Tab. 2. Differential gene expression in WT vs. 5xFADxhtau-KI at an age of 13 months. Supplementary Tab. 3. Applied antibodies. Supplementary Tab. 4. Applied Primers for DNA amplification and 3R/4R cDNA sequences. Supplementary Fig. 1. Differential gene expression analyzed using nCounter Mouse AD gene expression panel. Supplementary Fig. 2. 3R and 4R tau expression in dephosphorylated brain extracts analyzed by Western Blot. Supplementary Fig. 3. Gene Set Enrichment Analysis in 5xFADxhtau-KI mice at 7 months of age. Supplementary Fig. 4. Gene Set Enrichment Analysis in 5xFADxhtau-KI mice at 13 months of age. Supplementary Fig. 5. Plaque quantification in 7 and 13 months old 5xFAD and 5xFADxhtau-KI mice. Supplementary Fig. 6. Analysis of inflammatory phenotype in 5xFAD and 5xFADxhtau-KI. Supplementary Fig. 7. Comparison of distance and swim speed among genotypes. [file 13195_2022_1144_MOESM1_ESM.pdf]

## Supplementary information to

# A novel human tau knock-in mouse model reveals interaction of Abeta and human tau under progressing cerebral amyloidosis in 5xFAD mice

Susan Barendrecht, An Schreurs, Stefanie Geissler, Victor Sabanov, Victoria Ilse, Vera Rieckmann, Rico Eichentopf, Anja Künemund, Benjamin Hietel, Sebastian Wussow, Katrin Hoffmann, Kerstin Körber-Ferl, Ravi Pandey, Gregory W. Carter, Hans-Ulrich Demuth, Max Holzer, Steffen Roßner, Stephan Schilling, Christoph Preuss, Detlef Balschun, Holger Cynis

This file contains:

- Supplementary Tables 1-4
- Supplementary Fig. 1-7

## Supplementary Tables

**Supplementary Tab. 1** Differential gene expression in WT vs. 5xFAD at an age of 13 months. A rank-ordered list of differentially expressed genes. Orange-labeled genes are upregulated in 5xFAD. Analysis is based on n=4 of each genotype.

| mRNA | Log2 fold change | Std error (log2) | Lower confidence limit (log2) | Upper confidence limit (log2) | P-value | Probe.ID |
|---|---|---|---|---|---|---|
| Tyrobp | 1.69 | 0.216 | 1.26 | 2.11 | 5.83e-06 | NM_011662.2:130 |
| Fcer1g | 1.11 | 0.168 | 0.781 | 1.44 | 7.77e-05 | NM_010185.4:264 |
| C1qc | 1.11 | 0.169 | 0.779 | 1.44 | 7.77e-05 | NM_007574.2:708 |
| B2m | 0.818 | 0.126 | 0.572 | 1.06 | 7.77e-05 | NM_009735.3:340 |
| Ctss | 1.23 | 0.19 | 0.863 | 1.61 | 7.77e-05 | NM_021281.2:740 |
| Laptm5 | 0.994 | 0.155 | 0.69 | 1.3 | 9.21e-05 | NM_010686.3:36 |
| C1qa | 1.16 | 0.183 | 0.801 | 1.52 | 0.000102 | NM_007572.2:566 |
| Tmem176a | 0.703 | 0.113 | 0.481 | 0.926 | 0.000139 | NM_001098271.1:720 |
| Cyba | 0.99 | 0.161 | 0.675 | 1.31 | 0.000139 | NM_007806.3:135 |
| Lcp1 | 0.75 | 0.124 | 0.508 | 0.993 | 0.000172 | NM_001247984.1:3344 |
| C1qb | 1.11 | 0.186 | 0.747 | 1.48 | 0.000214 | NM_009777.2:865 |
| Tgfbr2 | 0.682 | 0.131 | 0.425 | 0.939 | 0.00241 | NM_009371.2:475 |
| Grn | 0.67 | 0.129 | 0.417 | 0.923 | 0.00241 | NM_008175.4:1292 |
| Rasgrp3 | 0.7 | 0.136 | 0.433 | 0.966 | 0.00254 | NM_001166493.1:940 |
| Tgfbr1 | 0.409 | 0.0817 | 0.249 | 0.569 | 0.00382 | NM_009370.2:4425 |
| Slco2b1 | 0.681 | 0.137 | 0.413 | 0.949 | 0.00383 | NM_175316.3:2720 |
| Csf1 | 0.48 | 0.0982 | 0.288 | 0.673 | 0.00479 | NM_001113530.1:833 |
| Itgb5 | 0.602 | 0.129 | 0.349 | 0.855 | 0.00959 | NM_001145884.1:1270 |
| Tmem176b | 0.464 | 0.101 | 0.267 | 0.661 | 0.0104 | NM_001164207.1:606 |
| Msn | 0.785 | 0.179 | 0.434 | 1.14 | 0.0199 | NM_010833.2:515 |
| Axl | 0.352 | 0.0803 | 0.194 | 0.509 | 0.0199 | NM_009465.3:3820 |
| Arhgdib | 0.528 | 0.122 | 0.289 | 0.767 | 0.0223 | NM_007486.4:280 |
| Pros1 | 0.569 | 0.133 | 0.308 | 0.831 | 0.026 | NM_011173.2:2720 |
| Arpc1b | 0.552 | 0.134 | 0.289 | 0.815 | 0.0394 | NM_023142.2:1291 |

**Supplementary Tab. 2** Differential gene expression in WT vs. 5xFADxhtau-KI at an age of 13 months. A rank-ordered list of differentially expressed genes. Blue-labeled genes are downregulated in 5xFADxhtau-KI, orange-labeled genes are upregulated in 5xFADxhtau-KI. Analysis is based on n=4 of each genotype.

| mRNA | Log2 fold change | Std error (log2) | Lower confidence limit (log2) | Upper confidence limit (log2) | P-value | Probe.ID |
|---|---|---|---|---|---|---|
| Mapt | -2 | 0.0667 | -2.13 | -1.87 | 3.13e-26 | NM_001038609.2:1202 |
| Tyrobp | 2.05 | 0.216 | 1.63 | 2.48 | 1.4e-08 | NM_011662.2:130 |
| Ctss | 1.45 | 0.19 | 1.08 | 1.83 | 2.53e-06 | NM_021281.2:740 |
| Fcer1g | 1.28 | 0.168 | 0.95 | 1.61 | 2.53e-06 | NM_010185.4:264 |
| Laptm5 | 1.16 | 0.155 | 0.854 | 1.46 | 3.37e-06 | NM_010686.3:36 |
| C1qc | 1.2 | 0.169 | 0.873 | 1.54 | 8.71e-06 | NM_007574.2:708 |
| Lcp1 | 0.864 | 0.123 | 0.622 | 1.11 | 1.1e-05 | NM_001247984.1:3344 |
| B2m | 0.868 | 0.126 | 0.622 | 1.11 | 1.3e-05 | NM_009735.3:340 |
| C1qa | 1.24 | 0.183 | 0.88 | 1.6 | 1.9e-05 | NM_007572.2:566 |
| Cyba | 1.08 | 0.161 | 0.763 | 1.39 | 2.02e-05 | NM_007806.3:135 |
| C1qb | 1.2 | 0.186 | 0.839 | 1.57 | 4.22e-05 | NM_009777.2:865 |
| Bex1 | -0.337 | 0.0538 | -0.443 | -0.232 | 7.51e-05 | NM_009052.2:620 |
| Tgfbr1 | 0.505 | 0.0816 | 0.345 | 0.665 | 8.72e-05 | NM_009370.2:4425 |
| Csf1 | 0.568 | 0.098 | 0.376 | 0.76 | 0.000306 | NM_001113530.1:833 |
| Tgfbr2 | 0.687 | 0.131 | 0.43 | 0.943 | 0.00175 | NM_009371.2:475 |
| Grn | 0.651 | 0.129 | 0.398 | 0.904 | 0.00316 | NM_008175.4:1292 |
| Tmem176a | 0.552 | 0.114 | 0.33 | 0.775 | 0.00536 | NM_001098271.1:720 |
| Pros1 | 0.643 | 0.133 | 0.382 | 0.904 | 0.00563 | NM_011173.2:2720 |
| Itgb5 | 0.602 | 0.129 | 0.349 | 0.856 | 0.00897 | NM_001145884.1:1270 |
| Slco2b1 | 0.629 | 0.137 | 0.361 | 0.897 | 0.0104 | NM_175316.3:2720 |
| Eno2 | 0.193 | 0.0434 | 0.108 | 0.278 | 0.0156 | NM_013509.2:1675 |
| Zbtb33 | -0.244 | 0.0557 | -0.353 | -0.134 | 0.0195 | NM_020256.2:1156 |
| Rasgrp3 | 0.58 | 0.136 | 0.313 | 0.848 | 0.027 | NM_001166493.1:940 |
| Lmo2 | 0.323 | 0.0767 | 0.172 | 0.473 | 0.03 | NM_001142335.1:824 |
| Srgn | 0.555 | 0.134 | 0.294 | 0.817 | 0.0333 | NM_011157.2:168 |
| Arpc1b | 0.539 | 0.134 | 0.276 | 0.802 | 0.0499 | NM_023142.2:1291 |

**Supplementary Tab. 3** Applied antibodies

| Antibody | Concentration/Dilution | | Product # | Manufacturer |
|---|---|---|---|---|
| | WB | IHC (DAB/IF) | | |
| **7E5** | 1µg/mL | - | 847-0102006301 | Analytik Jena |
| **T49** | 1:1000 | | MABN827 | Merck Millipore |
| **Tau13** | 1:200 | | Sc-21796 | Santa Cruz Biotechnology |
| **3R** | 1:1000 | 1:100 | 05-803 | Sigma Aldrich |
| **4R** | 1:2000 | 1:250 | CAC-TIP-4RT-P01 | Cosmo Bio |
| **MC1** | - | 1:50 | - | Peter Davies |
| **PHF1** | - | 1:250 | - | Peter Davies |
| **CP13** | - | 1:150 | - | Peter Davies |
| **A$\beta_{x\text{-}40}$** | - | 1:500 | 825201 | Biolegend |
| **Tau-5 (Pan-tau)** | 1:1000 | - | AHB0042 | Life technologies |
| **GAPDH** | 1:1000 | - | 21185 | Cell signaling |
| **Mouse IgG1** | - | 1:100 | 115-065-205 | Dianova |
| **Mouse IgG** | - | 1:100 | A16076 | Novex |
| **Rabbit IgG** | - | 1:100 | A16108 | Novex |
| **Cy3 rabbit IgG** | - | 1:500 | 111-165-144 | Dianova |
| **Cy2 mouse IgG** | - | 1:100 | 115-225-166 | Dianova |
| **HRP mouse IgG** | 1:2000 | - | 7076 | Cell signaling |
| **HRP rabbit IgG** | 1:1000/ 1:2000 | - | 7074 | Cell signaling |

**Supplementary Tab. 4** Applied Primers for DNA amplification and 3R/4R cDNA sequences

| Gene | Forward primer (5´-3´) | Reverse primer (5´-3´) |
|---|---|---|
| **Human 4R** | GAA GCT GGA TCT TAG CAA CG | GAC GTG TTT GAT ATT ATC CT |
| **Human 3R** | AGG CGG GAA GGT GCA AAT AG | TCC TGG TTT ATG ATG GAT GTT |
| **Mouse GAPDH** | ACT CCA CTC ACG GCA AAT TC | TCT CCA TGG TGG TGA AGA CA |
| **5xFAD (tg)** | CTA GGC CAC AGA ATT GAA AGA TCT | GTA GGT GGA AAT TCT AGC ATC ATC C |
| **5xFAD int. contr.** | AAT AGA GAA CGG CAG GAG CA | GCC ATG AGG GCA CTA ATC AT |
| **Human tau** | CTT GTC CCC AAC TCC ATA CC | GGA GAA CAC AGA CTG TGC TCC |
| **Mouse tau** | CTT GTC CCC AAC TCC ATA CC | ACT GCT TGA GTT ATC TTG GCC |
| **3R cDNA** | CCAGCCGGGAGGCGGGAAGGTGCAAATAGCTACAAACCAGTTGACCTGAGCAAGGTGACCTCCAAGTGTGGCTCATTAGGCAACATCCATCATAAACCAGGAGGTGGCCAG | |
| **4R cDNA** | AATTAATAAGAAGCTGGATCTTAGCAACGTCCAGTCCAAGTGTGGCTCAAAGGATAATATCAAACACGTCCCGGGAGGC | |

## Supplementary Figures

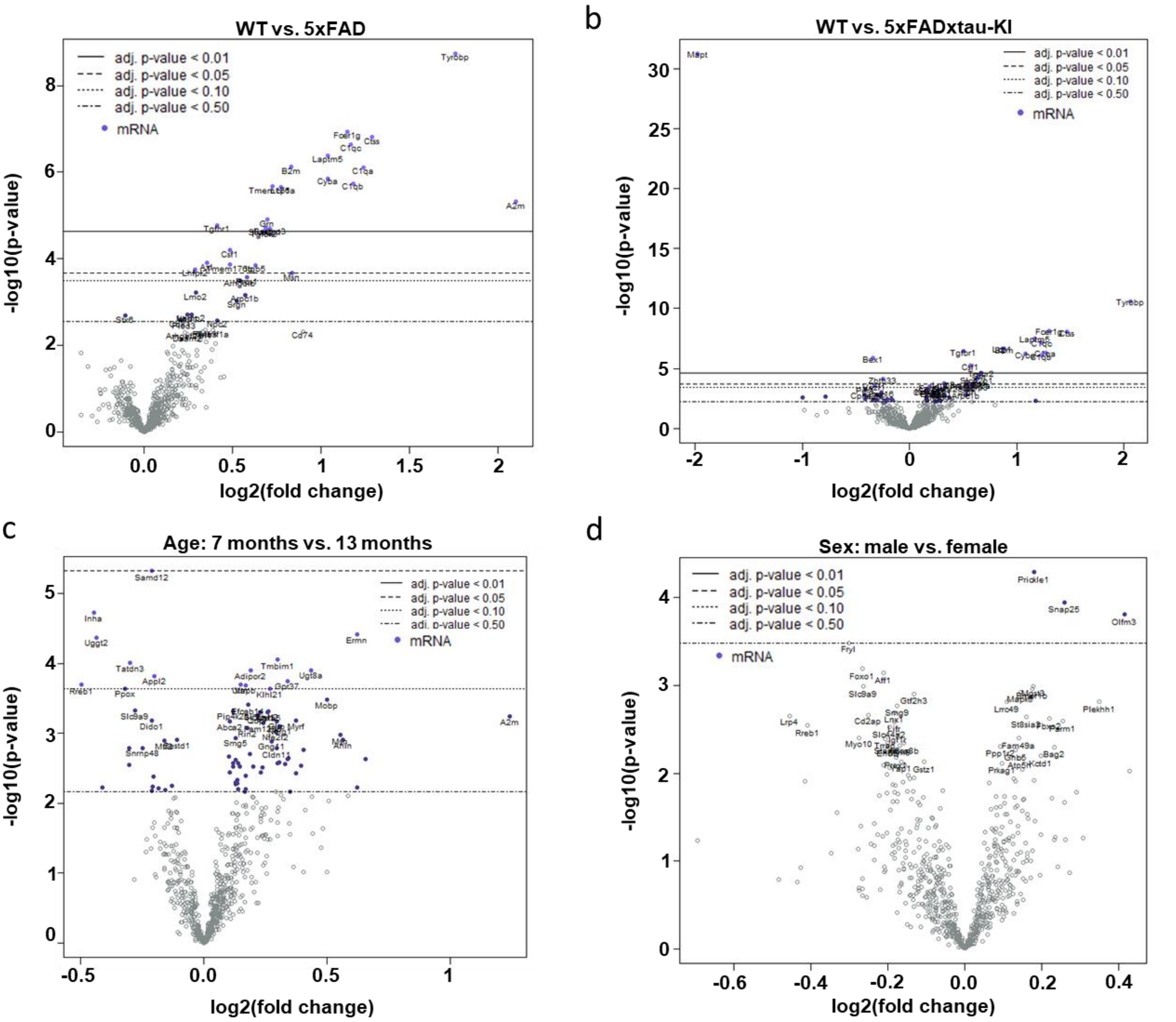


**Supplementary Fig. 1** Differential gene expression analyzed using nCounter Mouse AD gene expression panel. Depicted are Volcano plots showing log2 (fold change) in dependence of -log10 of *P*-values. **a**, Comparison of WT mice (n=12) and 5xFAD mice (n=12). **b**, Comparison of WT mice (n=12) and 5xFADxhtau-KI mice (n=12). **c**, Comparison of the age of 7 months vs. the age of 13 month (n=24 for each age). **d**, Comparison of males vs. females (n=24 for each sex).

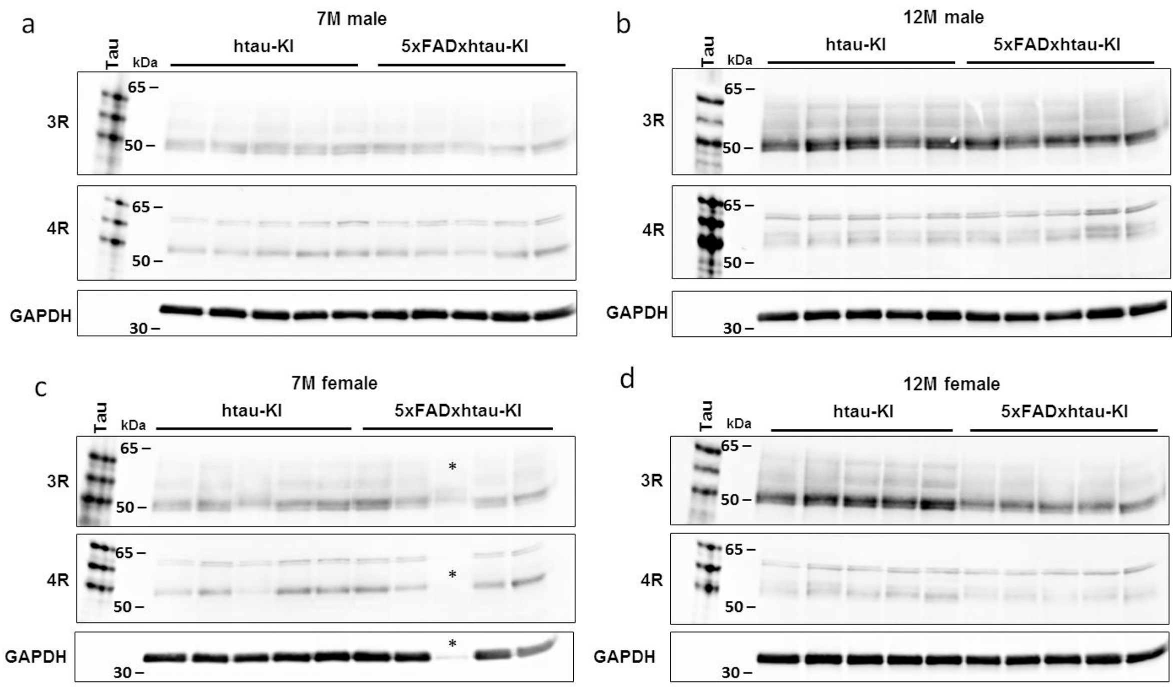


**Supplementary Fig. 2** 3R and 4R tau expression in dephosphorylated brain extracts analyzed by Western Blot. Western Blots of brain extracts for 3R and 4R tau from **a**, 7-month-old male, **b**, 12-month-old male, **c**, 7-month-old female, and **d**, 12-month-old female htau-KI and 5xFADxhtauKI mice. Nitrocellulose membranes were incubated with 3R-, 4R- and GAPDH-specific antibodies. Densitometric analysis of band intensities was performed using ImageJ with GAPDH as reference protein. The tau protein ladder was used as a standard as it contains 5 µg/ml of the six isoforms 0N3R, 1N3R, 2N3R, 0N4R, 1N4R, 2N4R each. n=4-5. *one sample was excluded from analysis due to inadequate loading of the gel.

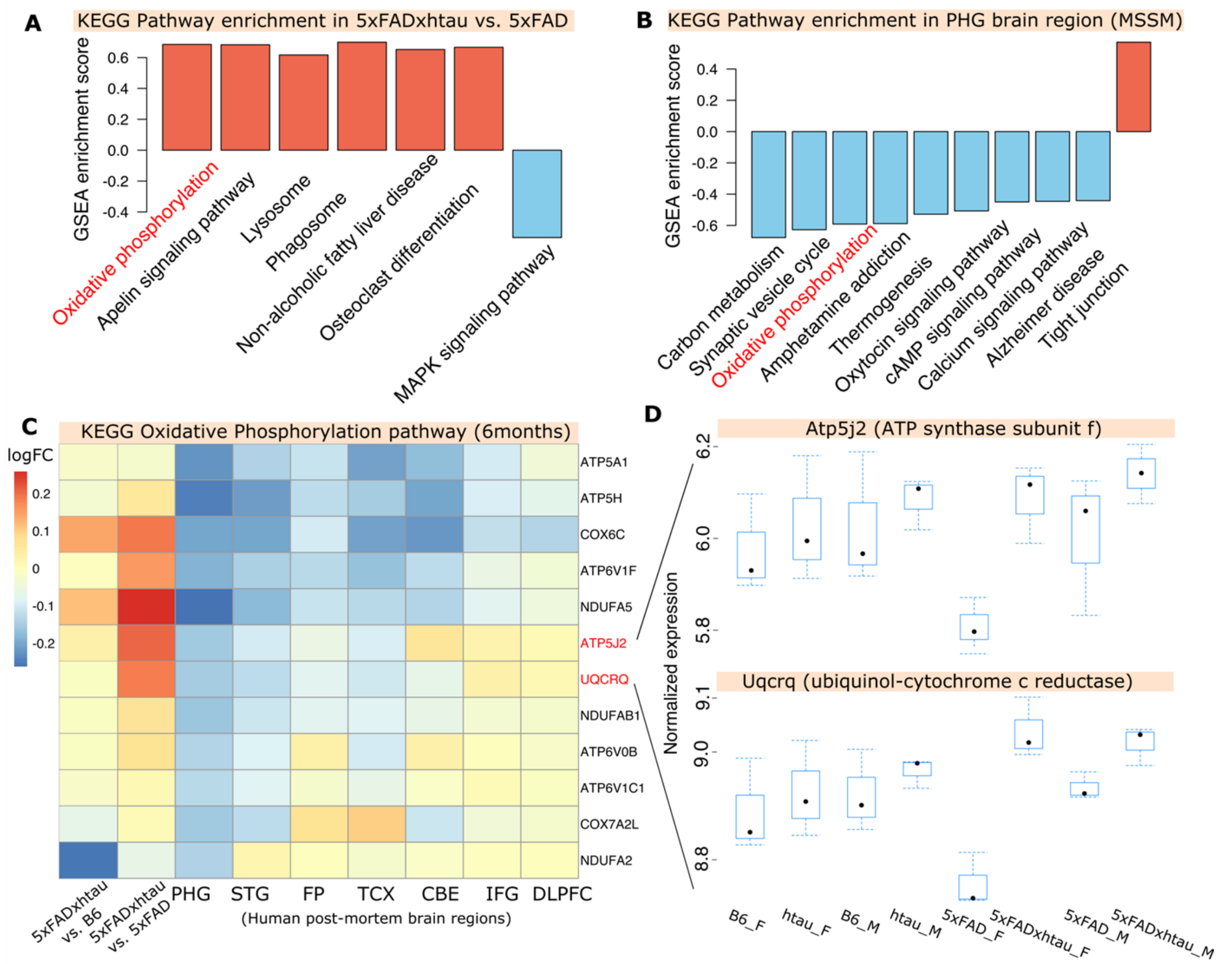


**Supplementary Fig. 3** Gene Set Enrichment Analysis in 5xFADxhtau-KI mice at 7 months of age. **a**, Gene Set Enrichment analysis of the NanoString panel at 7 months revealed a significant upregulation of multiple disease-associated pathways in 5xFADxhtau-KI mice compared to 5xFAD mice, including the oxidative phosphorylation and osteoclast pathways harboring multiple LOAD candidate genes. Oxidative phosphorylation was the most significantly associated pathway in 5xFADxhtau-KI mice compared to the 5xFAD mice. **b**, This pathway is downregulated in the parahippocampal (PHG) region of LOAD patients. **c**, Heat map showing the log fold change of oxidative phosphorylation pathway genes both in mouse transcripts compared to control mouse models and human transcripts of AD patients compared to control patients. **d**, Normalized expression of genes Atp5j2 and Uqcrq in female (F) and male (M) mice of wild type (B6), htau-KI (htau), 5xFAD and 5xFADxhtau-KI mice. n=6.

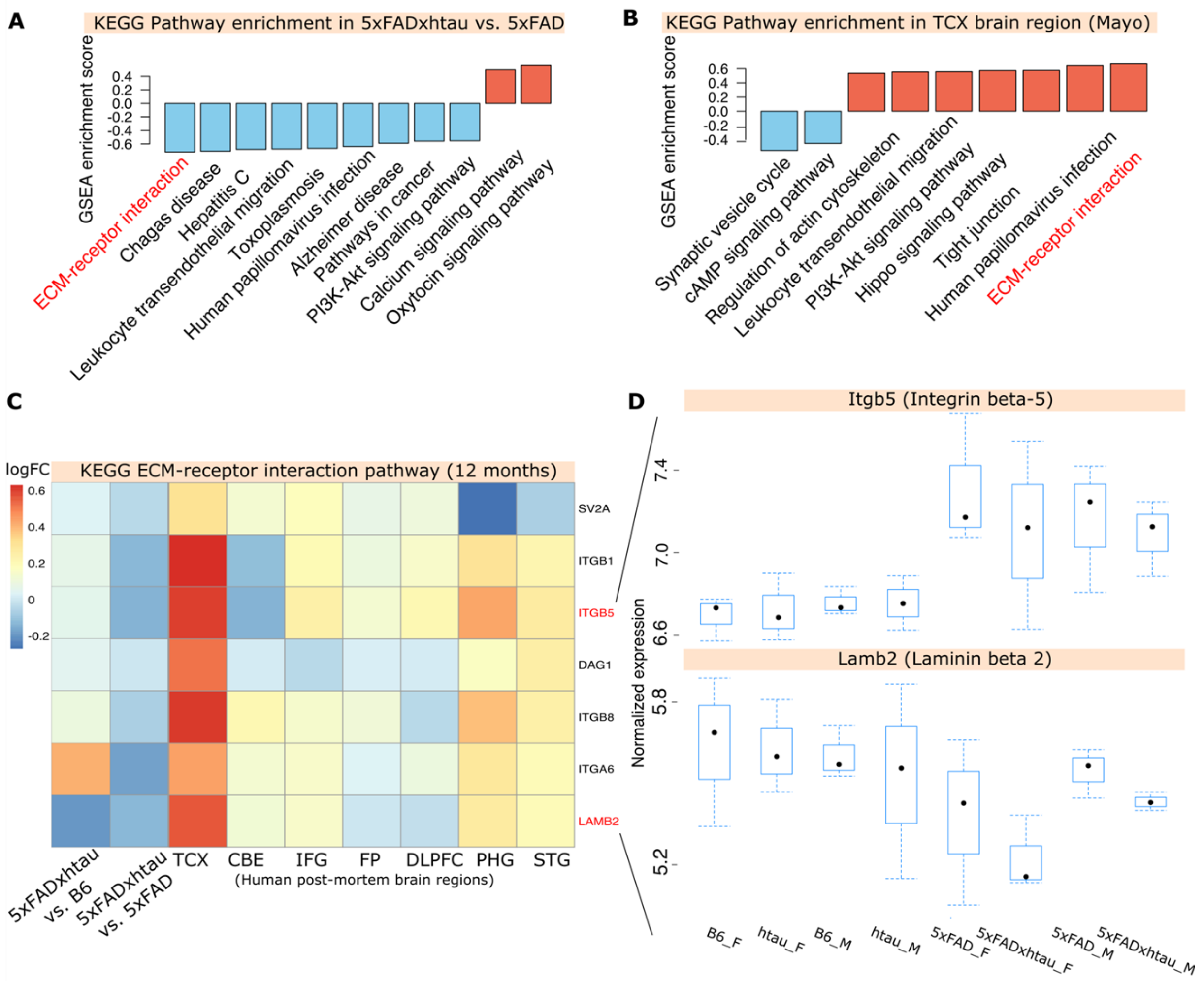


**Supplementary Fig. 4** Gene Set Enrichment Analysis in 5xFADxhtau-KI mice at 13 months of age. **a**, Gene Set Enrichment analysis of the NanoString panel at the age of 13 months revealed a significant downregulation of Extracellular matrix-receptor interaction (ECM-RI) and immune associated pathways in 5xFADxhtau-KI mice compared to 5xFAD mice. ECM-RI was the most significantly associated pathway in 5xFADxhtau-KI mice compared to the 5xFAD mice. **b**, This pathway is upregulated in the temporal cortex (TCX) of LOAD patients. **c**, Heat map showing the log fold change of ECM-RI pathway genes both in mouse transcripts compared to control mouse models and human transcripts of AD patients compared to control patients. **d**, Normalized expression of genes ITGB5 and LAMB2 in female (F) and male (M) mice of wild type (B6), htau-KI (htau), 5xFAD and 5xFADxhtau-KI mice. n=6.

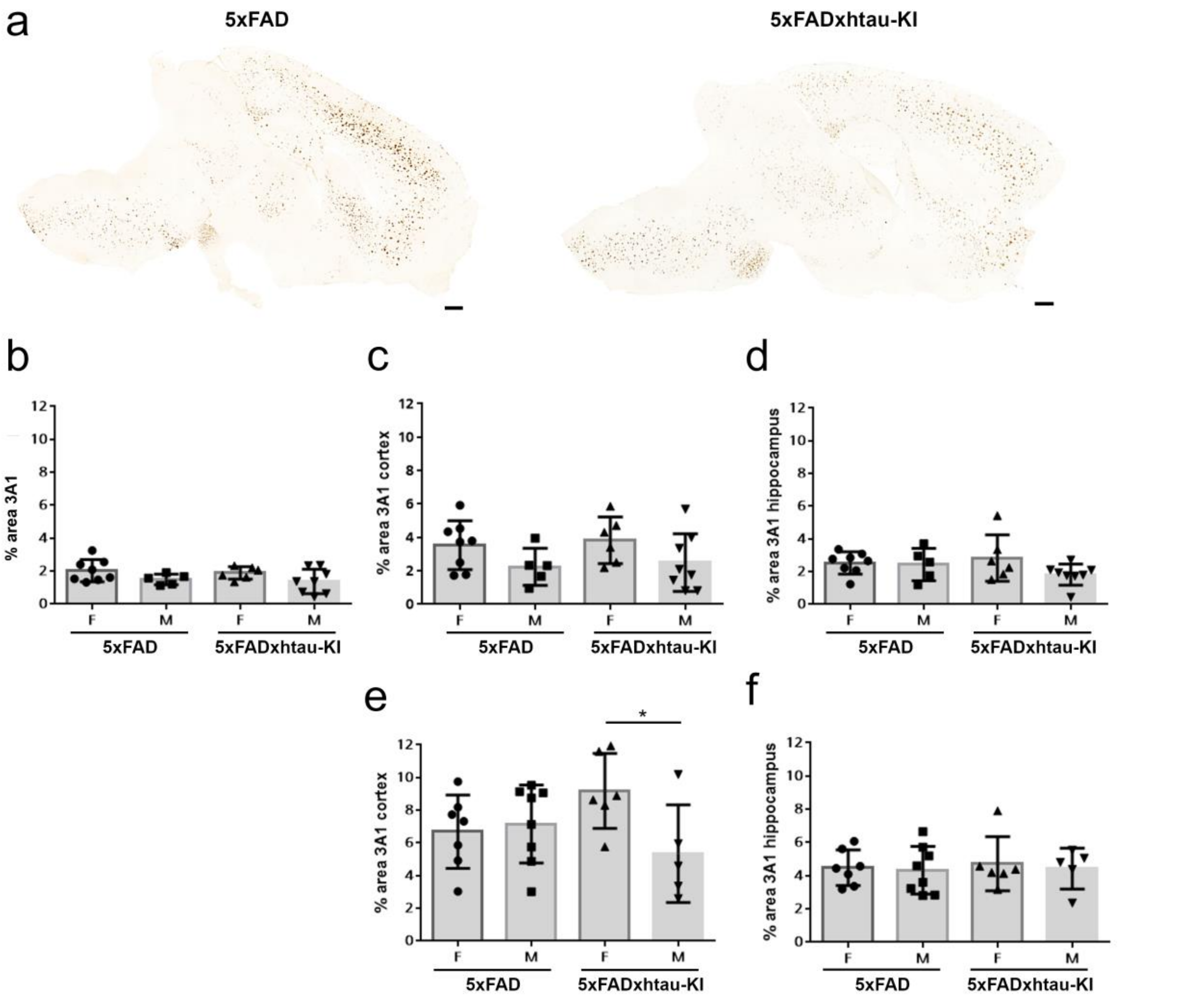


**Supplementary Fig. 5** Plaque quantification in 7 and 13 months old 5xFAD and 5xFADxhtau-KI mice. **a**, Representative sagittal brain slices of 7-month-old female 5xFAD and 5xFADxhtau-KI mice stained with 3A1 used for Aβ pathology quantification. Scale 500 µm. The occupied **b**, total **c**, cortical and **d**, hippocampal plaque area was further quantified. 3-5 slices per mouse and 5-8 mice per genotype and gender have been analyzed. Mean ± SEM. **e-f**, Data supplementary to Fig. 4c. Quantification of plaque area in 13-month-old 5xFAD and 5xFADxhtau-KI. Occupied **e**, cortical and **f**, hippocampal plaque areas are shown. Mean ± SEM. 3-5 slices per mouse and 5-8 mice per genotype and gender have been analyzed. * $p<0.05$. unpaired t-test. WT and htau-KI mice showed no plaque area (0%, data not shown).

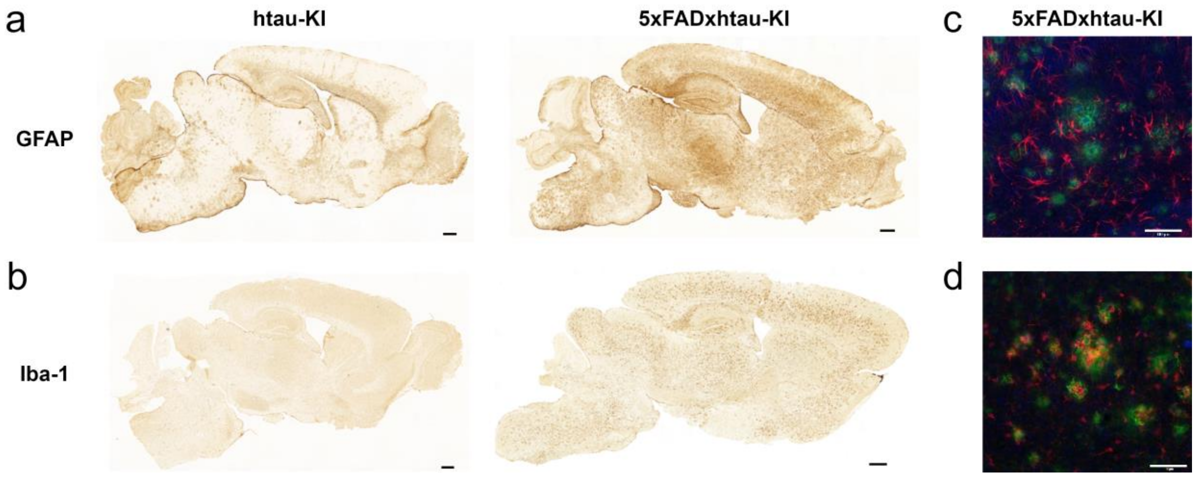


**Supplementary Fig. 6** Analysis of inflammatory phenotype in 5xFAD and 5xFADxhtau-KI. Representative sagittal brain slices of 13-month-old female htau-KI (left) and 5xFADxhtau-KI (right) stained for **a**, astrocytes (GFAP) and **b**, microglia (Iba-1). Clear microgliosis and astrocytosis were found in 5xFADxhtau-KI, whereas htau-KI showed no abnormal inflammatory phenotype. Scale 500 µm. **c**, Astrocytosis and **d**, microgliosis (were also visualized by immunofluorescence staining of plaques (3A1, green) relative to the distribution of immune cells (**c**, microglia, Iba1, red and **d**, astrocytes, GFAP, red) surrounding the plaques in 13-month-old 5xFADxhtau-KI mice. Scale 100 µm.

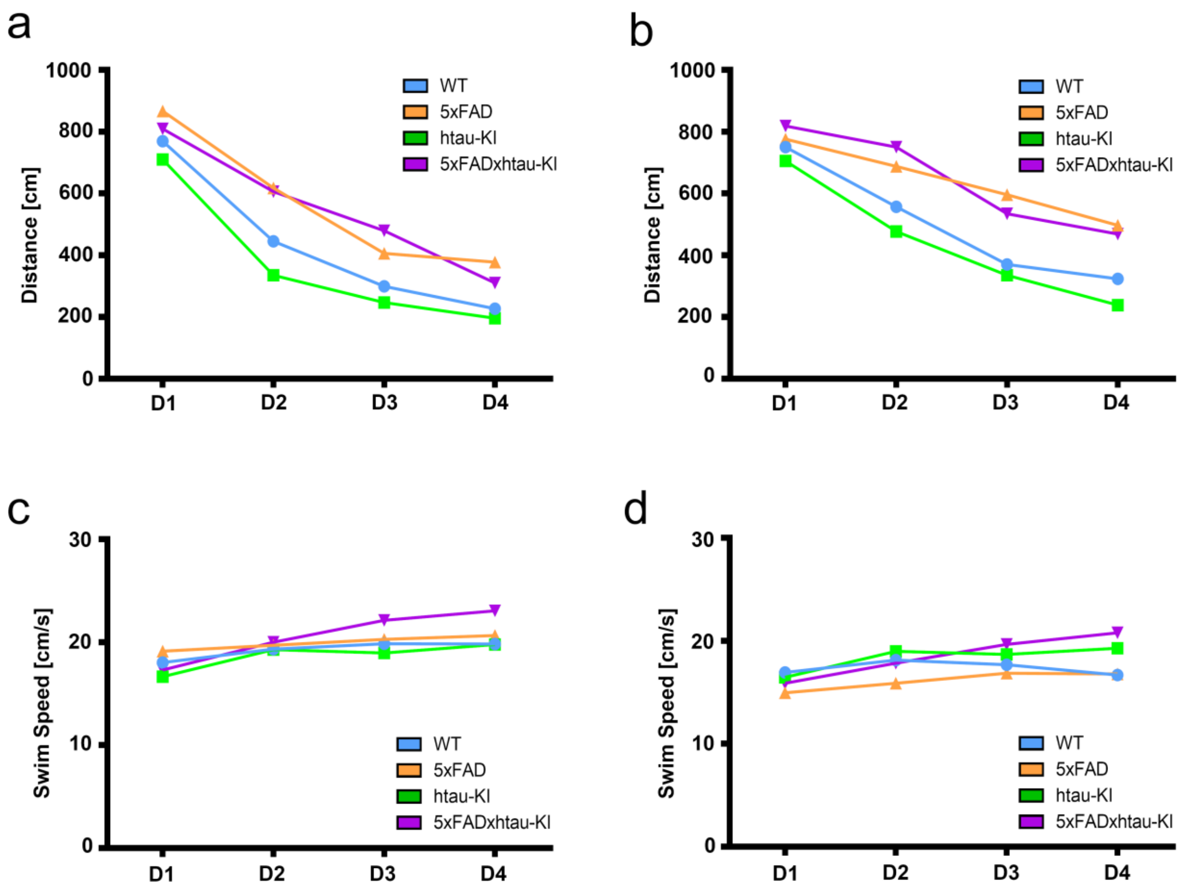


**Supplementary Fig. 7** Comparison of distance and swim speed among genotypes. Distance until finding of the platform in **a**, 6-month-old and **b**, 12-month-old mice. Distance is depicted in cm. Mean ± SEM. Swim speed in **c**, 6-month-old and **d**, 12-month-old mice was measured for each trial day separately. The speed was measured in cm/second. Mean ± SEM. For all figures: 6 months: WT (n=23), 5xFAD (n=24), htau-KI (n=21), 5xFADxhtau-KI (n=20) (male and female); 12 months: WT (n=24), 5xFAD (n=23), htau-KI (n=24), 5xFADxhtau-KI (n=19) (male and female).
