# Supplementary figures and images for: A novel human tau knock-in mouse model reveals interaction of Abeta and human tau under progressing cerebral amyloidosis in 5xFAD mice
Source: Alzheimers Res Ther. 2023 Jan 14;15:16. doi: 10.1186/s13195-022-01144-y (PMC9840277; doi:10.1186/s13195-022-01144-y)

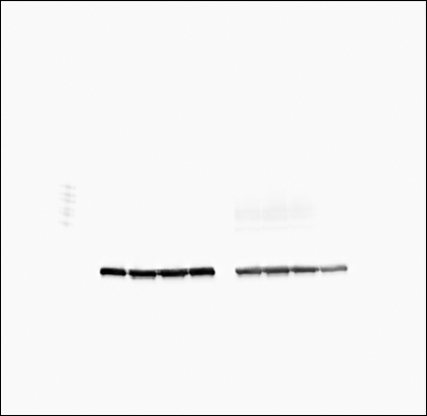

Supplement: Supplementary file 4 — Additional file 4. Original image file Figure 1b_GAPDH. [file 13195_2022_1144_MOESM4_ESM.tif]

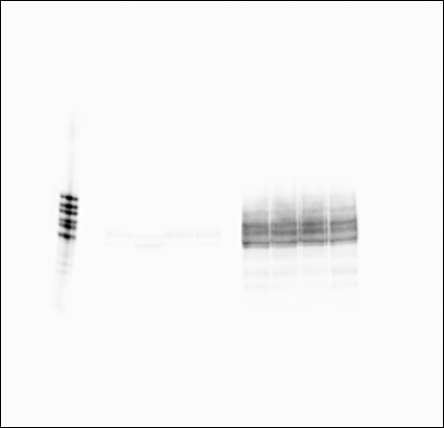

Supplement: Supplementary file 5 — Additional file 5. Original image file Figure 1b_Human tau. [file 13195_2022_1144_MOESM5_ESM.tif]

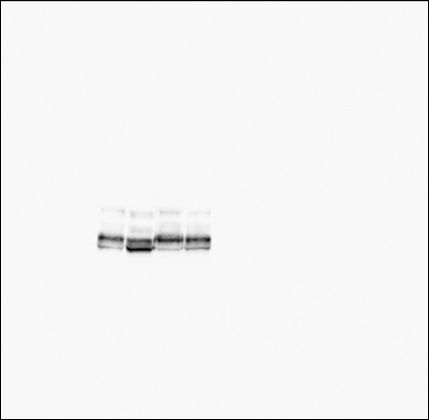

Supplement: Supplementary file 6 — Additional file 6. Original image file Figure 1b_Mouse tau. [file 13195_2022_1144_MOESM6_ESM.tif]

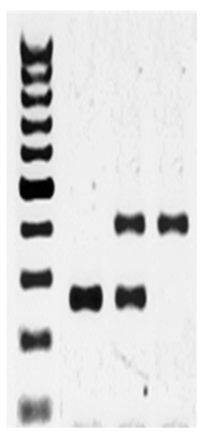

Supplement: Supplementary file 7 — Additional file 7. Original image file Figure 1d. [file 13195_2022_1144_MOESM7_ESM.tif]

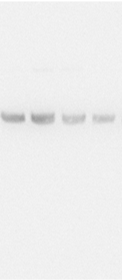

Supplement: Supplementary file 8 — Additional file 8. Original image file Figure 1e_GAPDH_htau-KI. [file 13195_2022_1144_MOESM8_ESM.tif]

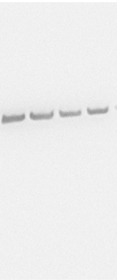

Supplement: Supplementary file 9 — Additional file 9. Original image file Figure 1e_GAPDH_WT. [file 13195_2022_1144_MOESM9_ESM.tif]

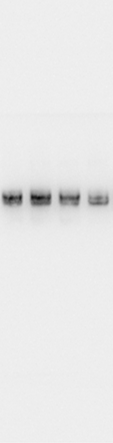

Supplement: Supplementary file 10 — Additional file 10. Original image file Figure 1e_total tau_htau-KI. [file 13195_2022_1144_MOESM10_ESM.tif]

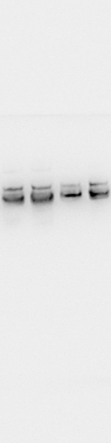

Supplement: Supplementary file 11 — Additional file 11. Original image file Figure 1e_total tau_WT. [file 13195_2022_1144_MOESM11_ESM.tif]

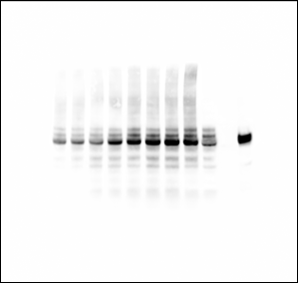

Supplement: Supplementary file 12 — Additional file 12. Original image file Figure 2e_3R. [file 13195_2022_1144_MOESM12_ESM.tif]

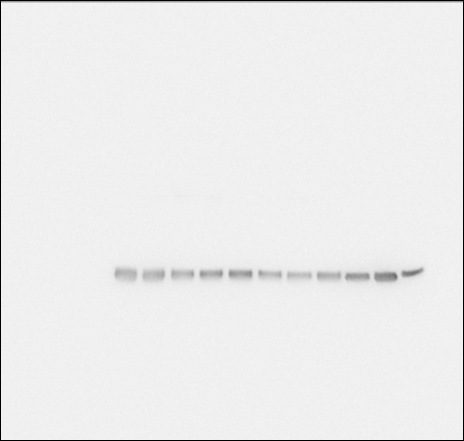

Supplement: Supplementary file 14 — Additional file 14. Original image file Figure 2e_GAPDH. [file 13195_2022_1144_MOESM14_ESM.tif]
